# Task-Driven Tailored Covalent Organic Framework for Dynamic Capture of Trace Radioactive CH_3_^131^I under High-Flow Rate Conditions

**DOI:** 10.1021/acscentsci.4c01318

**Published:** 2024-10-25

**Authors:** Linwei He, Baoyu Li, Zhonglin Ma, Fuqiang Zhao, Mingxing Zhang, Junchang Chen, Lingyi Li, Fangdong Tang, Linfeng He, Dongshuai Wu, Yadong Li, Lixi Chen, Long Chen, Chao Zhao, Kecheng Cao, Xing Dai, Zhifang Chai, Shuao Wang

**Affiliations:** † State Key Laboratory of Radiation Medicine and Protection, School of Radiation Medicine and Protection, Collaborative Innovation Center of Radiological Medicine of Jiangsu Higher Education Institutions, Soochow University, Suzhou 215123, China; ‡ Shanghai Institute of Measurement and Testing Technology, Shanghai 201203, China; § School of Physical Science and Technology & Shanghai Key Laboratory of High-resolution Electron Microscopy, ShanghaiTech University, Shanghai 201210, China

## Abstract

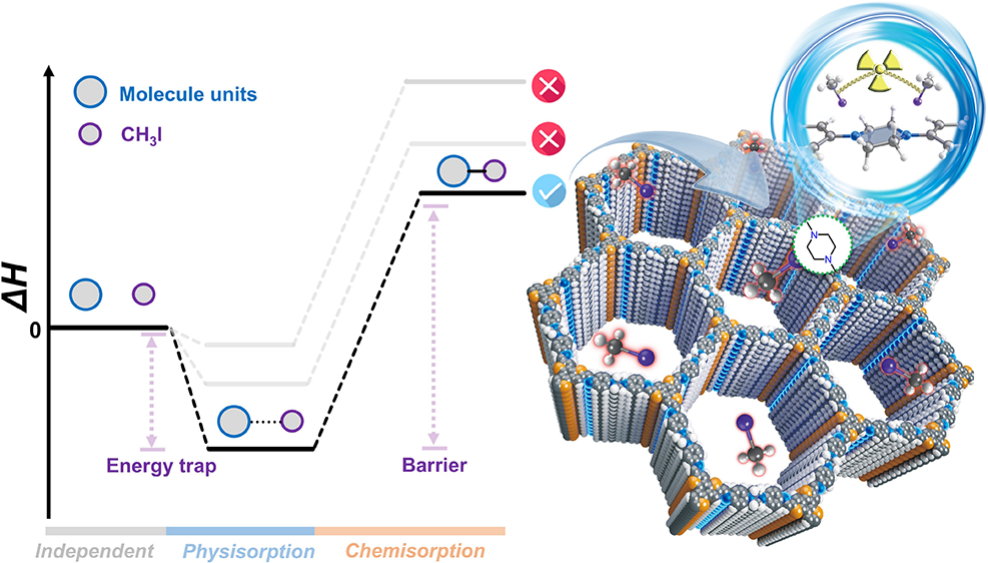

The removal of radioactive gaseous iodine is crucial for sustainable nuclear energy development, safe spent fuel management, and secure disposal of radioactive waste and radioactive medical waste. However, the efficient capture of gaseous iodine, particularly methyl iodide, under conditions of low concentration and high-flow rate that are representative of real-world scenarios remains underexplored. Herein, we adopted a “theory-first” strategy to design adsorbents with a superior affinity for methyl iodide. The rigorous theoretical calculations for both physisorption and chemisorption have guided us to rationally design a piperazine-based covalent organic framework material (Pip-COF, Pip = piperazine). The pioneering hot-testing under dynamic conditions, featuring low concentrations of 5 ppm radioactive CH_3_^131^I and a high-flow rate of 600 mL/min, demonstrated Pip-COF’s exceptional capture performance. Pip-COF exhibits saturated capacities of 39 mg/g at 75 °C and 78 mg/g at 25 °C, significantly outperforming the previously reported best COF (COF-TAPT, 6 mg/g at 25 °C) in this scenario. The gradual process of methylation and the identification of specific high-affinity sites were elucidated by time-resolved FT-IR spectroscopy and density functional theory (DFT) analysis, consistent with the design philosophy. This study exemplifies rational material design in facilitating the separation of trace pollutants in challenging environments.

## Abstract 2

Inspired by a “theory-first” paradigm, a piperazine-based covalent organic framework (Pip-COF) was designed to capture low-concentration CH_3_^131^I under high-flow rate.

## Introduction

Nuclear power, renowned for its remarkable energy density and low-carbon-emissions profile, ranks as one of the most significant contributors to low-carbon electricity generation.^1−3^ The operation of nuclear power stations and the reprocessing of spent nuclear fuel inevitably generate or release various radioactive isotopes, such as ^90^Sr, ^137^Cs, ^99^Tc, ^85^Kr, ^133^Xe, etc.^4^ Among these, radioactive iodine is a prominent component of fission products and a key contaminant in the environmental pollution and primarily exists in two isotopic forms: ^129^I (half-life, *t*_1/2_ = 1.57 × 10^7^ a) and ^131^I (half-life, *t*_1/2_ = 8.03 d).^5−8^ Radioiodine removal in reprocessing plants predominantly involves solution-based washing and solid-phase adsorption, with the latter being favored for its inherent simplicity, low energy consumption, and high efficiency.^5,9,10^ However, unlike the extensive research on elemental iodine removal, efforts to remove methyl iodide from off-gas streams during spent nuclear fuel dissolution have been limited.^5−8^ This challenge arises due to two factors: (1) the relatively stable molecular structure of CH_3_I makes it difficult to locate suitable sites on the adsorbent surface for strong adsorption and (2) organic iodides are present in much lower concentrations, accounting for only 0–10% of total gaseous radioiodine in the off-gas stream.^11−14^ Therefore, enhancing the binding affinity of CH_3_I for adsorbent active sites is essential to mitigate its leakage propensity.

Conventional approaches for removing organic iodide primarily involve either catalytic degradation with silver-based materials or *N*-methylation using nitrogen-rich adsorbents. Due to the high cost and potential contamination risks of silver,^15,16^*N*-methylation emerges as a more promising approach for developing economical and sustainable iodine capture systems.^17^ The design of functional adsorbents incorporating nitrogen-containing organic structures has opened up new horizons for gaseous methyl iodide capture^18^ but still remains in its infancy.

**Scheme 1:**
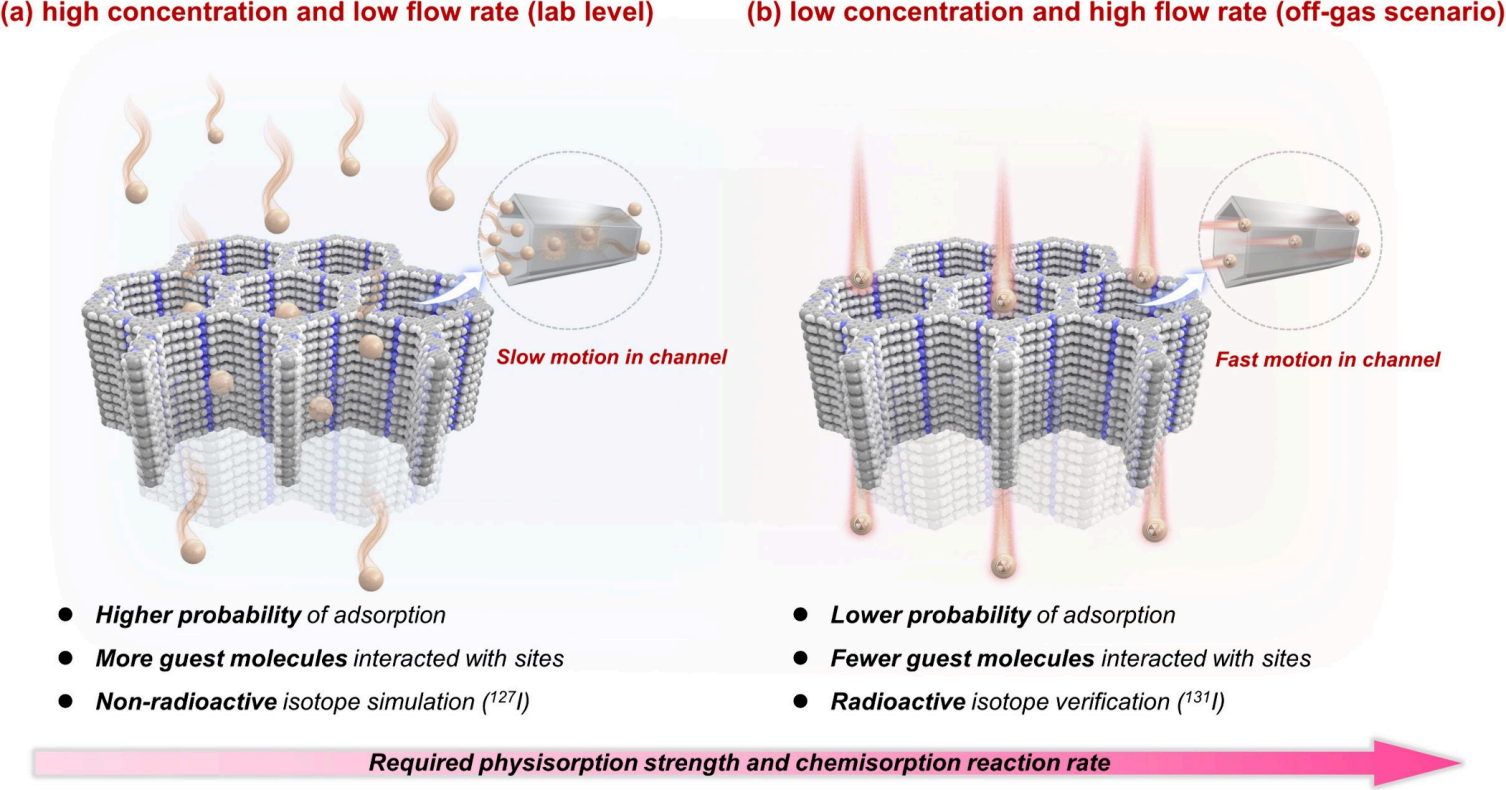
Differences between (a) the Previously Reported Dynamic Adsorption Conditions with High Concentration and Low Flow Rate on Lab Level and (b) the Optimized Dynamic Adsorption Simulated Conditions in This Study, Reflecting Lower Concentration and Higher Flow Rate, Better Aligning with Real-World Scenarios The structural skeleton represents typical COF adsorbent (host materials), and the yellow balls represent methyl iodide molecules (guest molecules). The markings on molecule balls represent radioactivity.

Covalent organic frameworks (COFs) represent cutting-edge crystalline organic polymers known for their customizable functionalities and porosities, facilitating task-specific guest capture.^19−27^ The innate organic composition of COFs lends them remarkable feasibility for the *N*-methylation strategy in CH_3_I removal. Recently, several COFs incorporating diverse nitrogen-containing functional groups have been synthesized for countering CH_3_I, preliminarily proving the potential of these materials for addressing the challenges associated with CH_3_I.^28,29^ However, most reported COFs have been tested under conditions of either static adsorption or dynamic adsorption characterized by high CH_3_I concentration (200000 ppm) and low flow rate (3 mL/min).^28−32^ In these scenarios, methyl iodide gas molecules traverse the COF channels at a measured pace, leading to a higher adsorption probability between CH_3_I molecules and active sites distributed within the channels (Scheme 1a). For more practical applications like off-gassing, the adsorption of radioactive CH_3_I at lower concentrations (50–5 ppm or less)^11,33^ and higher flow rates (200–800 mL/min)^34^ necessitates an improved binding affinity and adsorption rate between host materials and guest molecules (Scheme 1b).

The lack of data on the performance of COF in capturing trace CH_3_I in rapidly flowing off-gas emphasizes the need for innovative material design. However, the absence of a comprehensive quantitative analysis regarding the methylation propensity has resulted in the continued reliance on trial-and-error methods for fabricating high-performing COFs.^35^ How to rationally design efficient COFs for practical dynamic methyl iodide removal under high flow rate and ultralow concentration conditions remains an unsolved challenge. Herein, we propose a theory-guided paradigm to select appropriate N-containing moieties as building blocks in COFs, aimed at effectively addressing this issue effectively. By enhancing physisorption for CH_3_I capture and reducing *N*-methylation reaction barrier, a piperazine-tailored COF exhibits superior adsorptive properties compared to previously reported COFs for CH_3_I removal. This study sheds light on the vast potential of theoretically guided design in constructing high-performance adsorbents for environmental decontamination under harsh conditions.

## Results and Discussion

**Figure 1:**
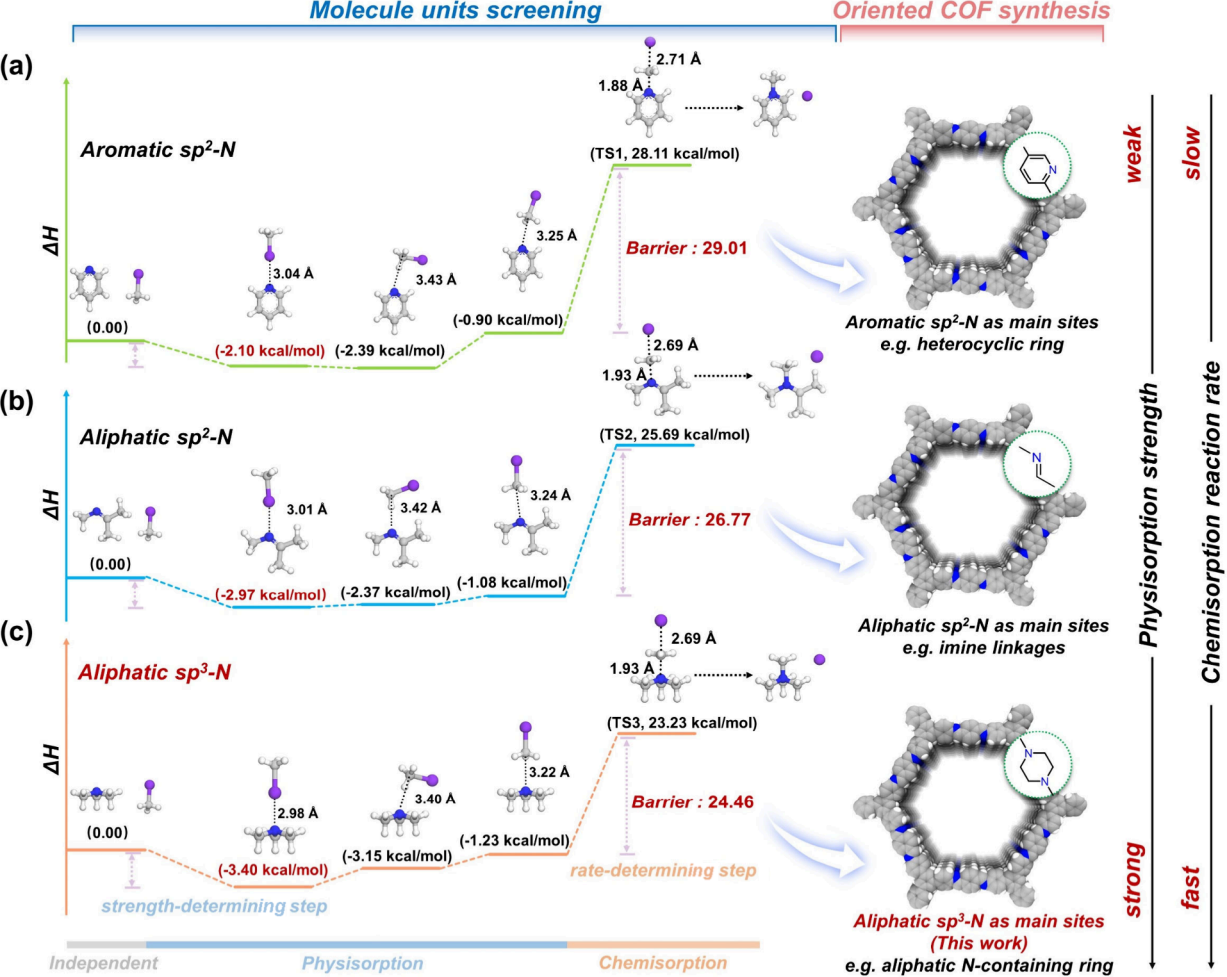
Theoretical screening for nitrogen-based functional units toward methyl iodide molecules. Diagram of CH_3_I adsorption on different screening units with transition state calculation of (a) aromatic sp^2^-N such as heterocyclic rings in COFs, (b) aliphatic sp^2^-N such as imine linkages in COFs, and (c) aliphatic sp^3^*-*N such as aliphatic N-containing rings in COFs. The corresponding models are representative chemical structural confirmations in previous studies and that proposed in this work. The color blocks are for the 3 processes of CH_3_I adsorption: gray for independent (interaction energy-free), blue for physisorption, and orange for chemisorption.

We first carried out quantum chemical calculations to screen nitrogen-based functional units (NFUs) that exhibit stronger affinity toward the CH_3_I molecule. Among numerous NFUs, we selected aromatic sp^2^-N (e.g., heterocyclic ring), aliphatic sp^2^-N (e.g., imine linkage), and aliphatic sp^3^*-*N (e.g., aliphatic N-containing ring) as representative theoretical models to evaluate their physisorption strength and the chemisorption reaction rate toward CH_3_I. Figure S1 displays the electrostatic potential (ESP) distributed on the electron density surfaces of CH_3_I and three NFUs. We observed that due to the classical nature of the carbon–halogen bond, both the CH_3_ group and the extension of the C–I bond display positive ESPs. Conversely, for NFUs, their N atoms exhibit prominent negative ESPs, which can be attributed to the presence of lone-pair electrons of N. As a result, N atoms of NFUs can serve as favorable sites to adsorb CH_3_I through strong N–CH_3_ and N–I electrostatic attractions. Subsequently, we fully considered the possible adsorption patterns during geometric optimizations and ultimately obtained three representative stable CH_3_I@NFU complexes for each NFU case, as shown in Figure S2. We found that CH_3_I can interact directly with the nucleophilic N sites of NFUs through the I atom or through CH_3_ “side-on” and CH_3_ “head-on” manners. To evaluate the physisorption strength of these NFUs toward CH_3_I, we calculated the corresponding interaction energy (*E*^int^) of CH_3_I with NFU in each CH_3_I@NFU complex. It is observed that the aliphatic sp^3^*-*N exhibits a relatively larger *E*^int^ compared to the cases of heterocyclic ring with aromatic sp^2^-N and imine linkage with aliphatic sp^2^-N. This suggests that CH_3_I can be more easily trapped in the adsorbent by introducing an aliphatic sp^3^*-*N group, such as an aliphatic N-containing ring, and maximizing their content, especially under a dynamic condition of low concentration and high-flow rate. Starting with isolated CH_3_I and NFUs as the initial substances, we next calculated the enthalpy changes (Δ*H*) of the *N*-methylation process for CH_3_I bound to the nucleophilic N sites of the NFUs. As shown in Figure 1, CH_3_I was first physically adsorbed to the N sites of NFUs through the I atoms or CH_3_ groups. These three physical adsorption conformations are very close in energy and can thus convert into each other through the rotations of the CH_3_I molecule. Moreover, aliphatic sp^3^*-*N exhibits a smaller activation barrier than the other two sp^2^-N cases (24.46 vs 26.77/29.01 kcal/mol), indicating that sp^3^*-*N has a faster *N*-methylation reaction rate and might be the most preferred chemisorption site. From these theoretical results, we found that sp^3^*-*N is the most outstanding adsorption site in both physisorption and chemisorption for CH_3_I. As a result, we considered incorporating this building unit into the COF framework to synthesize a novel COF material rich in aliphatic tertiary N atoms.

**Figure 2:**
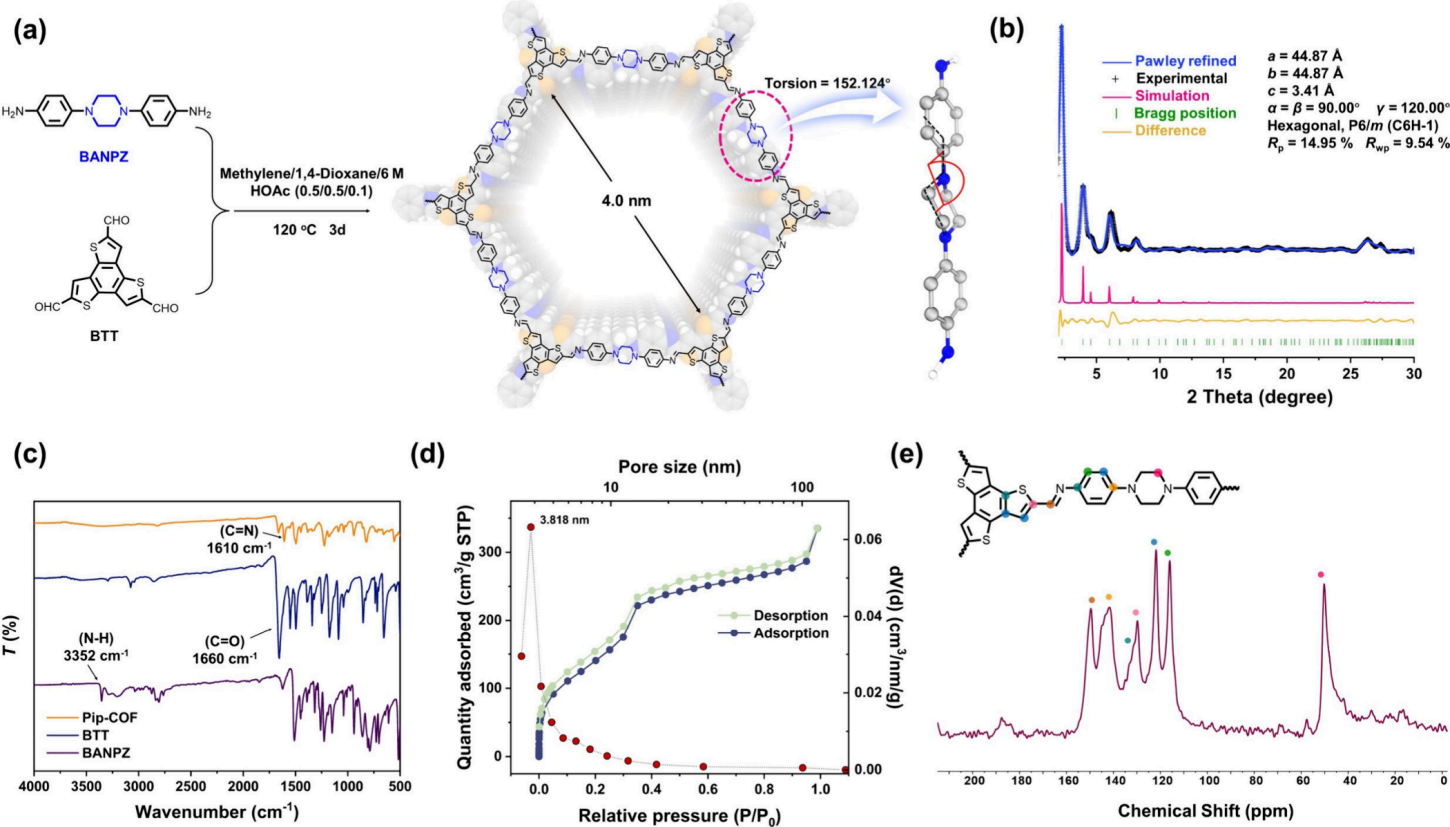
Structural simulations and characterizations of Pip-COF. (a) Synthetic route of Pip-COF with the structural diagram for a top view of Pip-COF. The highlighted inset illustrates the diagram of the piperazine moiety referring to the single-crystal structure of 1,4-bis(4-nitrophenyl)piperazine. (b) PXRD patterns of Pip-COF (black, experimental pattern; blue, simulated AA-eclipsed stacking model after Pawley refinement; pink, simulated AA-eclipsed stacking model; yellow, differences between experimental and simulation; green, Bragg positions of the simulated PXRD pattern of Pip-COF). (c) Fourier-transform infrared (FT-IR) spectra of Pip-COF, BTT, and BANPZ. (d) N_2_ adsorption isotherm for Pip-COF and corresponding pore size distribution (red dots). (e) Solid-state NMR ^13^C{^1^H} cross-polarization spectrum of Pip-COF.

Utilizing a theoretical screening of building blocks for CH_3_I capture and computational feasibility analysis for incorporating aliphatic tertiary N in COF skeletons, we rationally designed the piperazine-containing amino ligand 1,4-bis(4-aminophenyl)piperazine (BANPZ) to construct an ideal COF sorbent material with the desired adsorption performance under harsh operating conditions. Consequently, a piperazine-based COF named Pip-COF (Pip = piperazine) was successfully synthesized to serve as an expectant remover, mainly for subsequent methyl iodine adsorption (Figure 2a). The synthesis processes of ligand BANPZ and Pip-COF can be found in Figures S3–S5 in the Supporting Information.^36^ To characterize the crystallinity of the synthesized Pip-COF, powder X-ray diffraction (PXRD) was performed. As depicted in Figure 2b and Figure S9, the obvious signal peak at a low angle of 2θ = 2.33° suggests the presence of a macropore structure,^37,38^ corresponding to the (100) lattice plane. Furthermore, four minor peaks at 2θ = 3.9, 4.6, 6.2, and 8.1° are attributed to the (110), (200), (120), and (220) planes, respectively. The broad characteristic peak at a high angle of 25.4° suggests ordered interlayer stacking and the occurrence of π–π stacking effects.^39,40^ To further validate the rationality of the synthesized Pip-COF structure, a theoretical AA stacking model was constructed. Subsequently, after optimization with Pawley refinement, the model underwent a slightly expanded unit cell with parameters *a* = *b* = 44.866 Å, *c* = 3.412 Å, α = β = 90°, and γ = 120° and a symmetry of *P*6/*m*. The refined model demonstrates matching factors with experimental values of *R*_p_ = 14.95% and *R*_wp_ = 9.54%, confirming the congruence between our constructed theoretical model and the experimental structure of Pip-COF. The piperazine-based building unit, due to its nonconjugated nature, exhibits a chairlike spatial structure and provides greater electronegativity toward CH_3_I.

For further confirmation of successful Pip-COF synthesis, Fourier transform infrared (FT-IR) spectroscopy was conducted on Pip-COF and the two initial ligands (Figure 2c). In comparison to the ligands, the new signal peak observed around 1610 cm^–1^ of Pip-COF is assigned to the stretching vibration of C=N, thereby indicating the formation of the imine linkages.^41^ To verify the permanent porosity of Pip-COF, the N_2_ adsorption–desorption isotherm was collected at 77 K. As shown in Figure 2d, the rapid N_2_ uptakes in a comparatively low-pressure range (*P*/*P*_0_ < 0.05) demonstrate a typical isotherm of type IV assigned to the mesoporous characteristic of Pip-COF.^42^ The Brunauer–Emmett–Teller (BET) specific surface area was calculated to be 562.73 m^2^ g^–1^. Furthermore, the corresponding pore size distribution (PSD) analysis fitted with the Barrett–Joyner–Halenda (BJH) model indicates the main pore size of 3.818 nm, very close to the theoretical one of Pip-COF, which reinforces the robustness of the simulated AA-stacking model of Pip-COF. The solid-state NMR ^13^C{^1^H} cross-polarization spectrum further proved the incorporation of the designed structural moieties. Especially, the appearance of a characteristic signal at approximately 49 ppm, which is attributed to the methylene group on piperazine units, demonstrates its successful inlay in the framework of Pip-COF (Figure 2e).^28,29^

**Figure 3:**
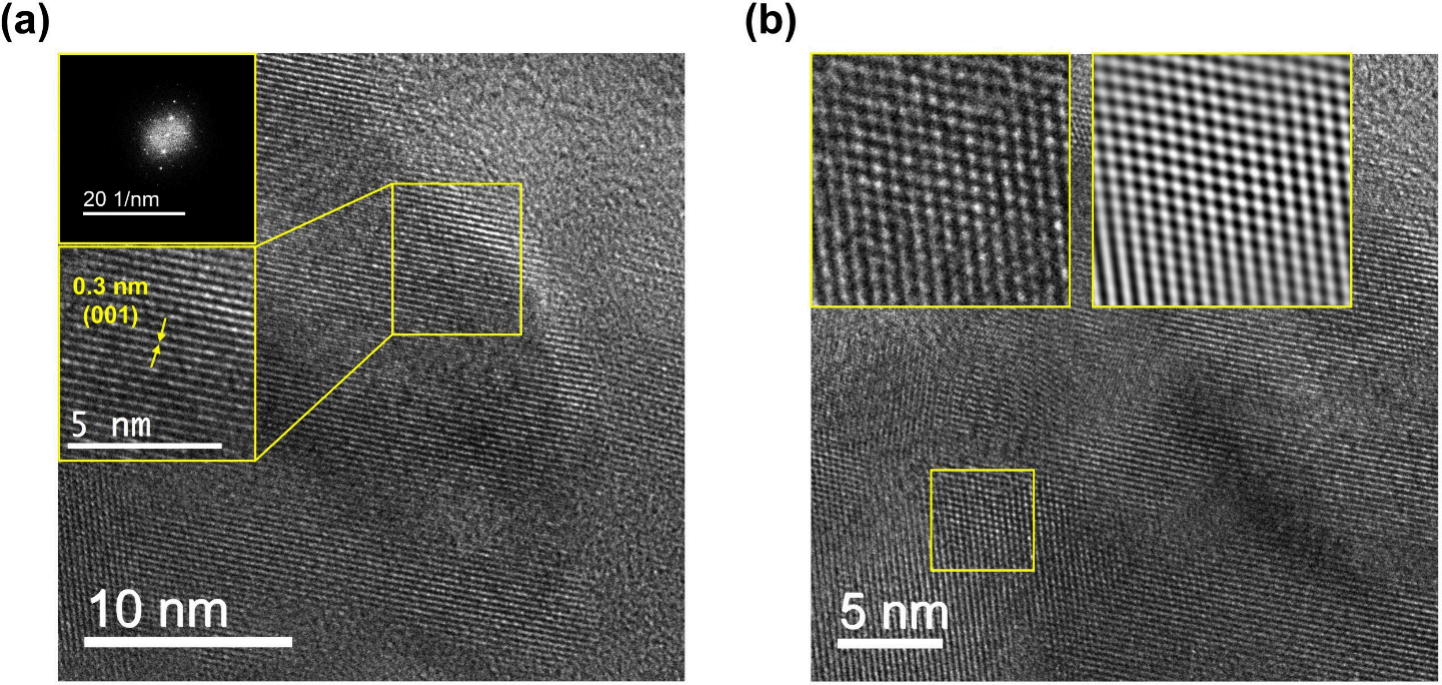
HRTEM characterization of Pip-COF. (a) High-resolution TEM image of Pip-COF (scale bar, 10 nm). Inset: corresponding fast Fourier transform (FFT) analysis and a magnified image of lattice structure from selected yellow area (scale bar, 5 nm). (b) High-resolution TEM image of Pip-COF (scale bar, 5 nm). Inset: the magnified image and corresponding Fourier-filtered image of selected yellow area.

More importantly, the periodic structural characteristics of Pip-COF are visualized by high-resolution transmission electron microscopy (HRTEM). As shown in Figure 3a,b, an interplanar spacing of 0.3 nm which is ascribed to 001 planes combined with the fast Fourier transform (FFT) analysis is observed, confirming the robust layered structure of Pip-COF. Thermogravimetric analysis (TGA) shows that Pip-COF retains its structural stability up to 400 °C, indicating that the sorbent can be used under scenarios at elevated temperatures (Figure S10).

**Figure 4:**
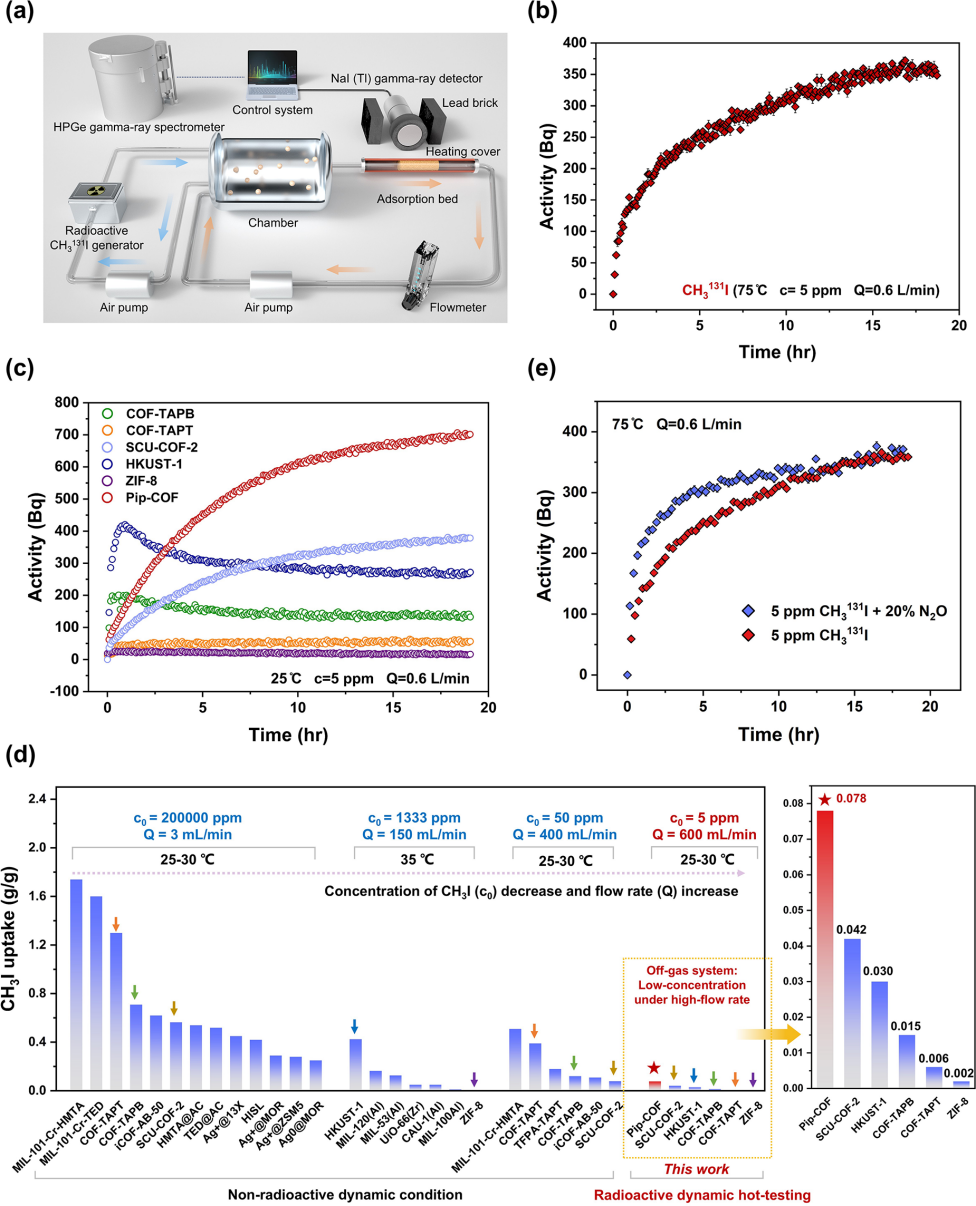
(a) Schematic model for the ^131^I column experiment with a gas circuit device and a real-time radiometric monitor. The detailed radioactivity measurement process and corresponding data collection are described in the Supporting Information. (b) Column adsorption results for CH_3_^131^I by Pip-COF under a simulated off-gas system at 75 °C. (c) Time-dependent adsorption performance of various sorbent materials including Pip-COF, COF-TAPB, COF-TAPT, SCU-COF-2, HKUST-1, and ZIF-8 at 25 °C. The initial concentration of CH_3_^131^I gas is 5 ppm and the flow rate is 0.6 L/min. (d) The systematic comparison of absolute adsorption capacity for CH_3_^131^I of per gram adsorbents by all of the column adsorbents for dynamic CH_3_I adsorption. The small arrows with same color represent the same column material tested in different systems. The highlighted portion for Pip-COF, COF-TAPB, COF-TTAPT, SCU-COF-2, HKUST-1 and ZIF-8 (25 °C; the initial concentration of CH_3_^131^I gas is 5 ppm and the flow rate is 0.6 L/min) tested in this work (related to Figure 4c and Table S1). (e) Effect of 20% N_2_O on adsorption performance for CH_3_^131^I by Pip-COF at 75 °C.

Motivated by the structural design philosophy, ^131^I dynamic hot-testing was subsequently performed within a dynamic adsorption test platform (Figure 4a), adapted from a metrological calibration system for airborne radioactive iodine monitoring^43^ and a gaseous radioactive iodine generator^44^ (refer to the Supporting Information for more details). This is the first ever radioiodine uptake experiment tested on a reticular framework material. The dynamic adsorption testing platform enables the execution of dynamic adsorption tests under specific conditions by incorporation of an adjustable iodine chamber. This chamber allows for customization of CH_3_I or I_2_ concentration, temperature, and NO_*x*_ concentration. Consequently, the platform facilitates the creation of the required testing conditions, notably by allowing for low concentrations and high flow rates to simulate real-world scenarios. The real-time monitoring of the dynamic adsorption process within the adsorption column was facilitated through a NaI(Tl) γ-ray detector, providing temporal data on the activity of radioactive iodine absorbed in the column. Subsequently, the accurate activity of radioactive iodine finally adsorbed was measured using an high-purity germanium (HPGe) gamma ray spectrometer. The conversion of the adsorbed masses of CH_3_I or I_2_ in the adsorption column from activity was carried out through decay correction considerations and specific activity (refer to the Supporting Information for more details).

The CH_3_^131^I removal by Pip-COF is shown in Figure 4b; the activity in the adsorption column continuously increased over time and tended to reach the adsorption equilibrium after about 20 h, finally achieving a capacity of nearly 350 Bq (0.039 g/g). The similar test for another species of iodine (^131^I_2_) was also performed and the final cumulative activity achieved 1520 Bq (0.169 g/g) (Figure S11). These above results preliminarily demonstrate that Pip-COF can effectively adsorb the two main gaseous iodine pollutants in an off-gas stream.

Based on the challenges in methyl iodide remediation and our predesigned philosophy on Pip-COF, we focus on CH_3_^131^I adsorption in subsequent dynamic experiments. To verify the advantages of Pip-COF compared to previously reported advanced column materials, we evaluated other several representative benchmark adsorbent materials, including (i) MOFs, such as ZIF-8^45−47^ (a stable zeolite imidazole-based MOF), and HKUST^47,48^ (a Cu-based MOF that has been demonstrated to be one of the column adsorbents toward multiple species of iodine gas) and (ii) COFs, such as SCU-COF-2^28^ (the first COF reported to capture CH_3_I), COF-TAPB, and COF-TAPT^29^ (two COFs for simultaneous capture of I_2_ and CH_3_I, showing the highest CH_3_I uptake under a high concentration of 200000 ppm in a dynamic system up to now). As determined by the dynamic adsorption results and the converted total adsorption capacity value (Figure 4c,d and Figure S12), Pip-COF shows the best performance with the highest enriched activity of 705 Bq (0.078 g/g) at 25 °C, remarkably exceeding SCU-COF-2 (378 Bq, 0.042 g/g) and COF-TAPB (134 Bq, 0.015 g/g) with weaker interactions based on nonaromatic sp^2^-N (imine) and aromatic sp^2^-N (pyridine or triazine) sites. In contrast, ZIF-8 (16 Bq, 0.002 g/g) and even COF-TAPT (55 Bq, 0.006 g/g) have almost no enrichment capacity toward low-concentration and high-flow CH_3_^131^I. Although HKUST-1 exhibits a considerable performance of 272 Bq (0.030 g/g), its accumulated activity partially decreases after reaching the peak value, which may correspond to a physiosorption effect. The dynamic adsorption results of Pip-COF at 25 °C exceed those at 75 °C, which is consistent with the phenomenon in previous studies.^29,31,32^

To comprehensively elucidate the performance characteristics of Pip-COF, we present a comparative analysis encompassing all column adsorbents previously documented for CH_3_I removal under diverse dynamic test conditions^13,28,29,47^ (Figure 4d, Table S1). Our analysis reveals a discernible trend wherein a decrease in the initial concentration of CH_3_I (*c*_0_) or an increase in flow rate (*Q*) leads to a significant reduction in the saturated dynamic CH_3_I uptake by the same adsorbent (e.g., SCU-COF-2, HKUST-1, and COF-TAPT). Given that the adsorption driving forces predominantly emanate from a multitude of weak interactions and channel effects, the adsorption capacities of static or dynamic systems with high concentrations and low flow rates prove inadequate for predicting adsorption performance in more practical scenarios. On contrast, owing to the rational theoretical screening, Pip-COF exhibits superior adsorption capacity compared with various representative COFs or MOFs, which underscores the critical role of enhancing binding affinity with CH_3_I for its dynamic removal in low concentrations.

Given the common occurrence of NO_*x*_ gas in off-gas systems (around 10000 ppmv, 1–4%),^5,34^ with a significant impact on traditional adsorbents such as Ag-based zeolites and activated carbon, the resistance to NO_*x*_ stands out as a critical parameter for appraising the viability of iodine adsorbents. Herein, we pioneered the adsorption performance for CH_3_^131^I under the NO_*x*_ atmosphere by COF material at 75 °C (for security consideration, N_2_O was chosen for coexisting gas in this experiment). Impressively, there is a nearly unparalleled ability to maintain its CH_3_^131^I uptake, even when exposed to 20% N_2_O gas simultaneously (initial CH_3_I concentration 5 ppm). This outstanding resistance to N_2_O by Pip-COF hints at potential applications in more intricate scenarios (Figure 4e).

**Figure 5:**
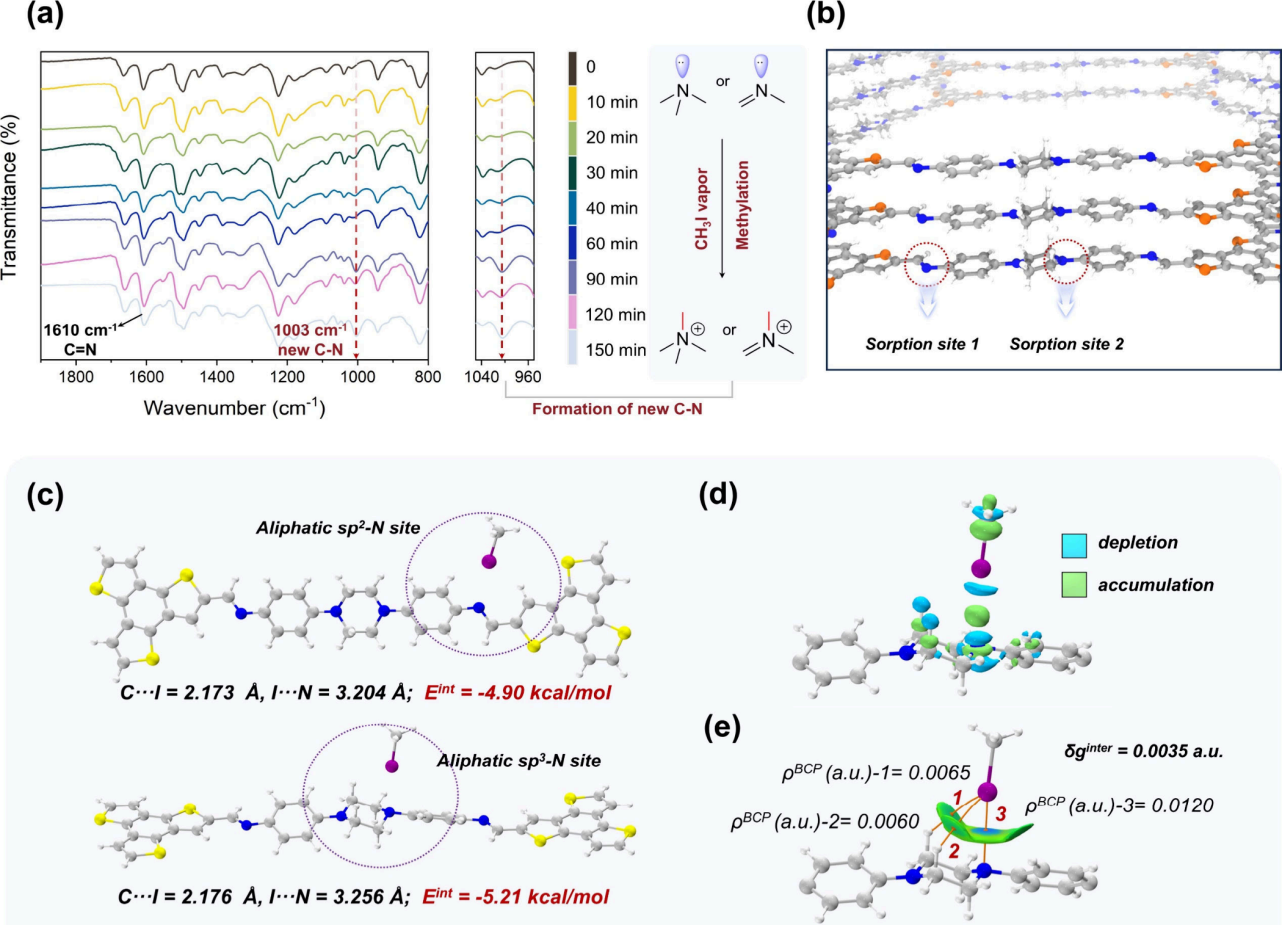
Mechanism for CH_3_I adsorption on Pip-COF. (a) Time-resolved FT-IR spectra of Pip-COF interacting with CH_3_I vapor at 75 °C. The magnifications reinforce the signals enhanced with the formation of new C–N from methylation. (b) Adsorption sites of CH_3_I molecules on Pip-COF. The magnifications display aliphatic sp^2^-N site (sorption site 1) and sp^3^-N site (sorption site 2), respectively. (c) DFT calculations of the binding energies (*E*^int^) of CH_3_I with different N sites of Pip-COF. (d) Electron density difference (EDD) isosurface maps plotted to illustrate the electron reorganization effects after CH_3_I coordinates to the sp^3^-N site of Pip-COF. The green and blue isosurfaces represent regions where the electron density increases and decreases, respectively. (e) Sign(λ^2^)ρ colored IGMH δg^inter^ = 0.0035 au isosurfaces. The blue and green colors represent strong attractive interaction and van der Waals interaction, respectively. The orange spheres and lines in the IGMH map represent the bond critical points (BCPs) and bond paths, respectively. The electron density values at the BCPs are labeled.

To further understand the main adsorption sites and species of iodine entrapped in Pip-COF, solid-state ^13^C NMR, FT-IR, and X-ray photoelectron spectroscopy (XPS) analyses were conducted. First, ^13^C NMR spectra convey the identical information on the carbon skeleton of Pip-COF after CH_3_I loading (Figure S13). As the pattern indicated, the characteristic peak at 50.2 ppm, assigned to the carbon atoms of the methylene group, exhibited a discernible broadening and slight shift with the loading of methyl iodide onto Pip-COF, suggesting the formation of carbon atoms on quaternary ammonium salt through methylation. In the FT-IR spectra, the bonds of C–N in piperazine (1382 cm^–1^) and C=N in imine (1610 cm^–1^) simultaneously decrease after the adsorption of CH_3_I or I_2_, implying that the two types of N atoms interact with iodine (Figure S14).^29,32,47^ To further reveal the process of CH_3_I adsorption, time-resolved FT-IR spectra were obtained with Pip-COF interacting with CH_3_I vapor from 0 to 150 min (Figure 5a). The increased peak at 1003 cm^–1^ in the magnification demonstrated the visible formation of new C–N bonds, originating from the methylation on 2 types of sorption sites on Pip-COF (Figure 5b).^29,31^ Moreover, the XPS analysis revealed the specific species of iodine in COF. As illustrated in Figure S15, compared to the spectra of pristine Pip-COF, two new characteristic peaks emerge at 618.1 and 629.9 eV, which are attributed to I 3d_5/2_ and I 3d_3/2_ originating from I^–^ (Figure S16). More importantly, the N 1s spectra reinforced the main interaction sites between the COF and CH_3_I molecule (Figure S17). For the as-synthesized Pip-COF, two obvious peaks at 398.9 and 399.6 eV are collected, which correspond to the nitrogen atoms from imine and piperazine groups, respectively. After adsorption, a new split peak emerges with higher binding energy at 402.02 eV, demonstrating the formation of nitrogen cations from the original neutral nitrogen atoms.^13,29,49^ The more pronounced variation in peak area observed for N atoms in piperazine underscored its heightened affinity for CH_3_I, in accordance with our preliminary theoretical screening.

Encouraged by the preliminary conjecture on sorption sites and data from spectral characterization, density functional theory (DFT) calculations were performed to study the host–guest interactions of CH_3_I with Pip-COF. A one-layered fragment that contains the foremost structural features was selected to simulate the local structure of Pip-COF. Figure 5c shows the stable physical adsorption patterns of CH_3_I with Pip-COF, and it can be seen that both sp^2^-N and sp^3^-N can serve as adsorption sites for CH_3_I through N–I electrostatic attractions. The calculated interaction energies (*E*^int^) of CH_3_I with sp^2^-N and sp^3^-N were −4.90 and −5.21 kcal/mol, respectively, suggesting that CH_3_I can be more easily captured by sp^3^-N of the Pip-COF adsorbent. To gain further insights into the electron reorganization effects upon adsorption, we plotted the electron density difference (EDD) isosurface map for the case of the sp^3^-N–CH_3_I complex. As shown in Figure 5d, when the I atom of CH_3_I interacts with sp^3^-N, significant electron transfer and polarization are observed. In the region between I and N, electron densities decrease (blue) near N and I and increase (green) between N and I, indicating that the electron densities of both N and I simultaneously polarize to the I–N bond region. This reveals the pseudoelectron sharing feature between N and I caused by the I–N interaction. Moreover, the sp^3^-N–I interaction was further investigated using the independent gradient model based on Hirshfeld partition (IGMH). As shown in Figure 5e, the green and blue regions of the IGMH isosurface indicate van der Waals (vdW) interaction and a prominent attractive weak interaction (such as a halogen bond), respectively. It can be seen that the notable halogen bond between the I atom of CH_3_I and the N atom of Pip-COF is distinctly unveiled by the light blue region in the IGMH isosurface. Except for the I–N interaction, there is an infinitely extended green isosurface between the I atoms and the H atoms of piperazine, which clearly shows notable vdW interactions.

## Conclusion

In summary, with the guidance of theoretical screening, we successfully synthesized a piperazine-based covalent organic framework and investigated its removal properties for CH_3_^131^I via radiological hot-testing. Owing to the judicious selection of an aliphatic sp^3^-N moiety characterized by increased binding affinity, Pip-COF exhibits exceptional adsorption efficacy for CH_3_^131^I at the parts per million level coupled with elevated flow velocity, surpassing various representative column adsorbents operating under identical conditions. Crucially, the consistent stability in the adsorption amount for CH_3_^131^I even in the presence of N_2_O gas underscores its robustness in complex environments. This work unequivocally confirms the high superiority of this task-specific strategy, which heralds a new paradigm for the design and fabrication of high-performance adsorbents tailored for environmental remediation.

## Supporting Information Available

Materials and methods and computational and experimental details (PDF)
Crystallographic data (CIF)

## Supplementary Material

oc4c01318_si_001.pdf

oc4c01318_si_002.cif
