# Supplementary material for: Task-Driven Tailored Covalent Organic Framework for Dynamic Capture of Trace Radioactive CH3131I under High-Flow Rate Conditions
Source: ACS Cent Sci. 2024 Oct 25;10(11):2072–81. doi: 10.1021/acscentsci.4c01318 (PMC11613281; doi:10.1021/acscentsci.4c01318)
Supplement: Supplementary file 1 — oc4c01318_si_001.pdf [file oc4c01318_si_001.pdf]

# Supporting Information

## Task-Driven Tailored Covalent Organic Framework for Dynamic Capture of Trace Radioactive $CH_3{}^{131}I$ under High-Flow Rate Conditions


Linwei He[1†], Baoyu Li[1†], Zhonglin Ma[1†], Fuqiang Zhao[1], Mingxing Zhang[1], Junchang Chen[1], Lingyi Li[1], Fangdong Tang[2], Linfeng He[2], Dongshuai Wu[2], Yadong Li[3], Lixi Chen[1], Long Chen[1*], Chao Zhao[2*], Kecheng Cao[3], Xing Dai[1], Zhifang Chai[1] and Shuao Wang[1*]

[1] *State Key Laboratory of Radiation Medicine and Protection, School of Radiation Medicine and Protection, Collaborative Innovation Center of Radiological Medicine of Jiangsu Higher Education Institutions, Soochow University, Suzhou 215123, China*

[2] *Shanghai Institute of Measurement and Testing Technology, Shanghai 201203, China*

[3] *School of Physical Science and Technology & Shanghai Key Laboratory of High-resolution Electron Microscopy, ShanghaiTech University, Shanghai 201210, China*

†These authors contributed equally.

E-mail: chenlong3@suda.edu.cn; zhaoc@simt.com.cn; shuaowang@suda.edu.cn.

**Experimental Section**

**Chemicals and reagents**. Piperazine (>99.5%, Macklin), 4-nitrofluorobenzene (>98%, Macklin) were directly purchased from Shanghai Macklin Biochemical Technology Co., Ltd. Benzobis[1,2-B:3,4-B':5,6-B']trithiophene-2,5,8-trialdehyde (BTT, 98%, Jilin Yanshen) was obtained from Jilin Yanshen Scientific Co., Ltd. Hydrazine monohydrate (analytical grade, National Pharmaceutical Group Corporation), potassium carbonate ($K_2CO_3$, analytical grade, National Pharmaceutical Group Corporation), N,N'-dimethylformamide (DMF, analytical grade, National Pharmaceutical Group Corporation), ethanol (EtOH, analytical grade, National Pharmaceutical Group Corporation), palladium on carbon catalyst (Pd/C, 97%, Leyan), iodomethane ($CH_3I$, 99.5%, with stabilizing agent copper filings, TCI), and deionized water were all purchased through commercial channels and were not further purified before use. $N_2$ (purity 99.999%) was purchased from Suzhou Lind Gas Co., Ltd. Radioactive $^{131}I_2$ was prepared by oxidizing and reducing non-radioactive NaI labeled with radioactive $^{131}I$ using $Fe_2(SO_4)_3$. The yield was 93% (20 min), and the initial concentration of $^{131}I_2$ was 0.81 ppm with a specific activity of 214 Bq/mg. Due to severe iodine deposition, the concentration of $^{131}I_2$ in the iodine chamber decreased rapidly over time. Radioactive $CH_3{}^{131}I$ was prepared through isotopic exchange between non-radioactive $CH_3I$ and radioactive NaI labeled with $^{131}I$. The yield was nearly 100%, and the initial concentration of $CH_3{}^{131}I$ was 5.07 ppm. Since methyl iodide had minimal deposition, the concentration of $CH_3{}^{131}I$ in the iodine chamber decreased over time primarily due to the adsorption process during the experiment. Safety Statement: radioactive elemental iodine ($^{131}I_2$) and methyl iodide ($CH_3{}^{131}I$) gases are volatile radioactive gases that emit β-rays (191.6 keV, 89.6%) and γ-rays (364.5 keV, 81.5%) during decay. Inhaling these gases poses severe health risks. All experiments involving $^{131}I$ in this research work were conducted in non-sealed radioactive material operation areas with radiation safety licenses.

**Instrumental Analysis Methods**. $^1H$ nuclear magnetic resonance ($^1H$ NMR) spectroscopy was performed on a DD2-600 (600 MHz) liquid-state superconducting NMR spectrometer produced by Agilent Technologies (China) Co., Ltd. PXRD patterns were collected from 2° to 30° at a 5° $min^{-1}$ scanning speed with a Bruker D8 Advance diffractometer with Cu Ka radiation (λ = 1.54056 Å) and a Lynxeye one-dimensional detector. The solid-state NMR $^{13}C\{^1H\}$ cross polarization spectrum were recorded on a Bruker Avance III WB-400 instrument. The Fourier transform infrared spectroscopy (FT-IR) spectra in the range of 4,000 to 400 $cm^{-1}$ region were acquired on a Thermo Nicolet iS50 spectrometer. NaI(Tl) gamma ray detector: a commercial NaI(Tl) scintillation crystal severed as the real-time gamma ray detector. The dimensions of the NaI(Tl) scintillation crystal were a cylinder with a height and diameter of 5.8 cm each. High-purity germanium (HPGe) gamma ray spectrometer: the HPGe gamma ray spectrometer was produced by Canberra Industries Inc., USA, with a detector diameter of 69.60 mm, and thickness of 31.30 mm.

**Computational Methods.** All quantum chemical calculations were carried out using the Gaussian 09 program[1]. Three N-based functional units ($CH_3I$), aromatic $sp^2$-N such as heterocyclic ring, aliphatic $sp^2$-N such as imine linkage and aliphatic $sp^3$-N such as trimethylamine were selected as the theoretical models to study their physisorption and chemisorption towards $CH_3I$. Geometric optimizations were performed at the PBE0-D3(BJ)/Def2TZVPPD level[2,3]. The electrostatic potential (ESP) on the van der Waals (vdW) surfaces (isodensity = 0.001 a.u.) of $CH_3I$ and $CH_3I$ were calculated based on their optimized structures. In order to evaluate the physisorption strength of three nitrogen-based functional units (NFUs) towards $CH_3I$, the interaction energies ($E^{int}$) were calculated at the ωB97xD/Def2QZVPPD[4,5] level using the following formula:

$$E^{int} = E(CH_3I@NFU) - E(CH_3I) - E(NFU)$$

where $E(CH_3I@NFU)$, $E(CH_3I)$ and E(NFU) represent the total energies of each $CH_3I$@NFU complex, $CH_3I$ fragment of $CH_3I$@NFU and NFU fragment of $CH_3I$@NFU, respectively. The basis set superposition error (BSSE) was corrected using the counterpoise correction method proposed by Boys and Bernardi[6]. To investigate the chemisorption of $CH_3I$ on NFUs, the enthalpy changes ($\Delta H$) for $CH_3I$ specifically bound to the nucleophilic N sites of NFUs through N-methylation reactions were calculated. The Enthalpy thermal corrections produced by frequency analysis were added to the single point energies to yield relatively accurate $H$ energy of various conformations. The electron density difference (EDD) and the independent gradient model based on Hirshfeld partition (IGMH) analysis were carried out using the Multiwfn program[15].

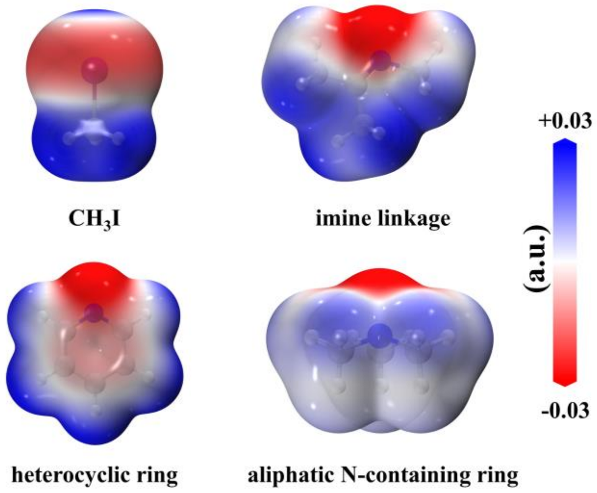


**Figure S1.** ESP distribution on the electron density surface (isodensity = 0.001 a.u.) of the $CH_3I$ and three NFUs.

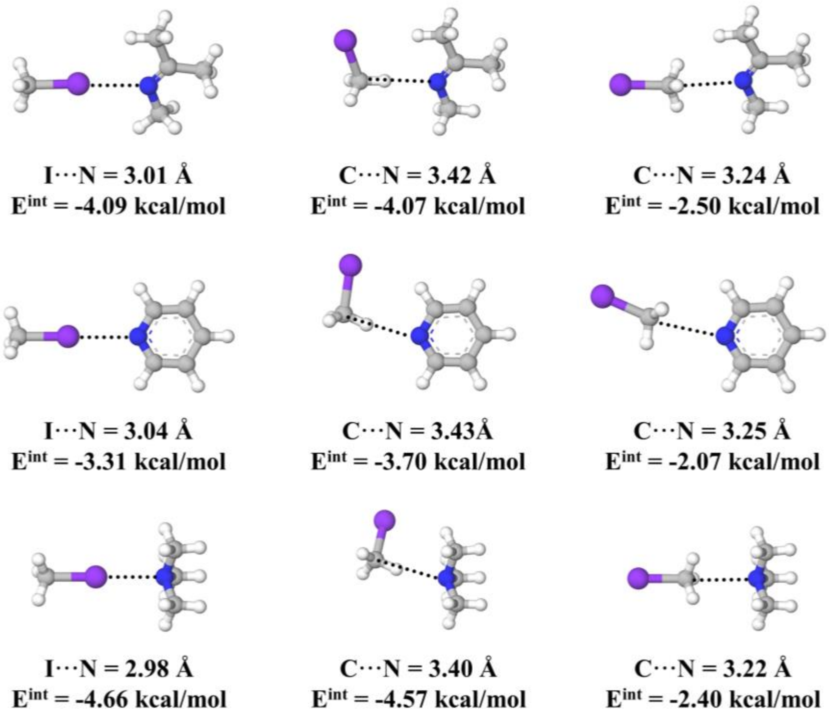


**Figure S2.** Optimized structures of $CH_3I$@NFU complexes. The I-N or C-N distances in each $CH_3I$@NFU complex are labeled by dot-dashed lines. $E^{int}$ denotes the interaction energy of $CH_3I$ with NFU.

**Synthesis of 1,4-bis(4-nitrophenyl)piperazine.**

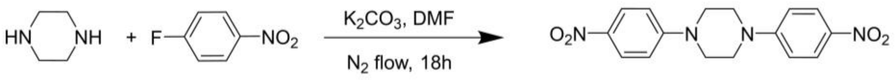


**Figure S3.** Synthesis of 1,4-Bis(4-nitrophenyl)piperazine

8.64 g (0.10 mol) of piperazine, 26.64 g (0.22 mol) of 4-fluoro-1-nitrobenzene, and 16.59 g (0.12 mol) of potassium carbonate were added to a 500 mL round-bottom three-neck flask. Subsequently, 150 mL of dimethylformamide (DMF) was added to the flask. The round-bottom flask containing the solid-liquid mixture was placed on a 140°C oil bath reactor, and the system was evacuated under liquid nitrogen cooling, followed by nitrogen purging, repeated three times. The reaction mixture was stirred and heated for 18 hours, then cooled to around 50°C. The resulting mixture was poured into ice-cold ethanol for recrystallization. The precipitated solid product was collected by suction filtration using a Buchner funnel and washed repeatedly with ethanol and deionized water. The solid product was dried in a vacuum oven at 90°C overnight, yielding orange-red crystals with a yield of approximately 86.2%.

**Synthesis of 1,4-bis(4-aminophenyl)piperazine (BANPZ).**

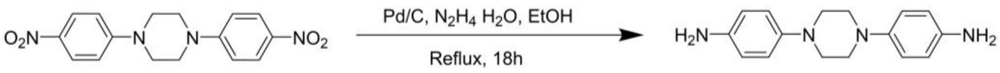


**Figure S4.** Synthesis of 1,4-bis(4-aminophenyl)piperazine (BANPZ).

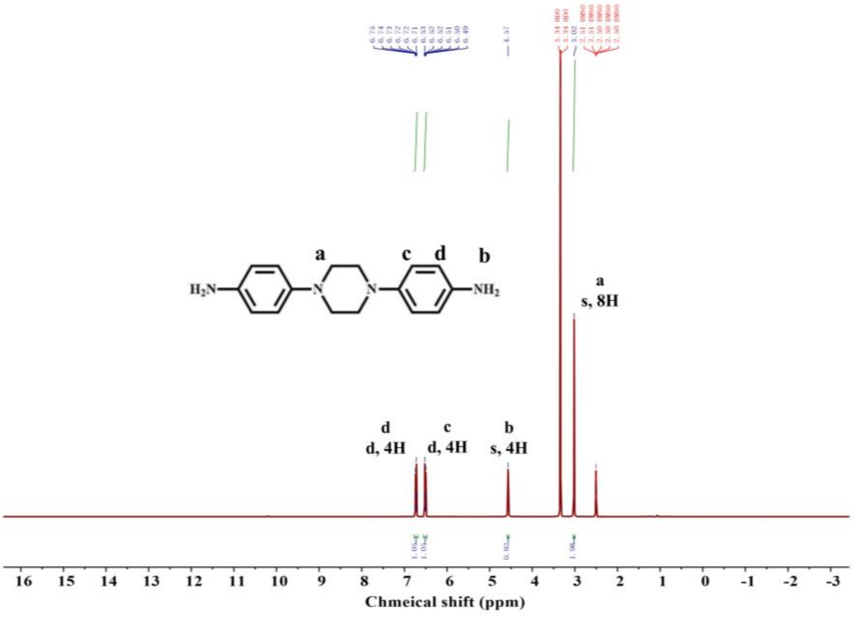


**Figure S5.** $^1$H NMR of 1,4-bis(4-aminophenyl)piperazine (BANPZ) in DMSO-$d_6$.

As illustrated in Figure S4, 13.13 g (0.04 mol) of the previously synthesized 1,4-bis(4-nitrophenyl)piperazine, 0.35 g (0.22 mol) of Pd/C catalyst, and 350 mL of ethanol were added to a 1000

mL three-neck round-bottom flask. Subsequently, 21 mL of hydrazine hydrate was slowly added to the reaction mixture in the round-bottom flask under a nitrogen atmosphere over approximately 2 hours. After 8 hours of reflux heating, the crude product was filtered to remove Pd/C and other solid impurities. The obtained filtrate was cooled under a nitrogen atmosphere. The precipitated solid crude product was collected by suction filtration and recrystallized using ethanol as the solvent. The recrystallized product was dried in a vacuum oven overnight, yielding a light pink final product with a yield of approximately 39.5%. The $^{1}$H nuclear magnetic resonance spectrum is shown in Figure S5 (DMSO-$d_6$, ppm, 400 MHz): δ (ppm) = 6.75 (d, 4H), 6.53 (d, 4H), 4.57 (s, 4H), 3.02 (s, 8H).

**Synthesis of Pip-COF.** The previously synthesized BANPZ ligand (12.06 mg, 0.045 mmol) and benzo[1,2-B:3,4-B':5,6-B']trithiophene-2,5,8-trialdehyde (BTT) (9.91 mg, 0.03 mmol) were added to a 10 mL Pyrex tube, followed by the addition of trimethylbenzene and 1,4-dioxane (0.5 mL: 0.5 mL) as solvents. The solid-liquid mixture was subjected to ultrasonication for 10-15 minutes until well dispersed. To the dispersion, 0.1 mL of 6 M acetic acid solution was added as an acid catalyst under a nitrogen atmosphere. The tube was sealed and subjected to three freeze-pump-thaw cycles under liquid nitrogen cooling. The reaction tube from the third thaw was placed in a constant temperature oven at 120°C and allowed to react for 3 days. After completion of the reaction, the tube was cooled to room temperature. The solid product at the bottom of the tube was collected and washed with tetrahydrofuran (THF) at least three times to remove oligomers and unreacted ligands. The washed product was dried in a vacuum oven at 60°C overnight. The dried solid was activated using a supercritical carbon dioxide apparatus with ethanol as the solvent, followed by additional drying to yield the final brown-yellow Pip-COF (Pip = piperazine) sample.

**Preparation of Radioactive $^{131}I_2$.** Radioactive $Na^{131}I$ solution was prepared by mixing a non-radioactive NaI reactant solution with a high-activity $Na^{131}I$ mother liquor. Gaseous $^{131}I_2$ was primarily prepared via the oxidation-reduction reaction of $Na^{131}I$ under oxidizing conditions[8,9]:

$$2\ Na^{131}I + Fe_2(SO_4)_3 + H_2O \rightarrow {}^{131}I_2\ (g) + 2FeSO_4 + Na_2SO_4$$

The specific activity of the radioactive $^{131}I_2$ was designed to be 180 Bq/mg at the moment of mixture serving as the reference time point.

**Preparation of Radioactive $CH_3{}^{131}I$.** Liquid radioactive iodomethane is prepared by the isotopic exchange between radioactive $Na^{131}I$ and non-radioactive $CH_3I$. Subsequently, it is vaporized into gaseous radioactive iodomethane and used in adsorption experiments. In order to ensure the activity measurements comparable across all adsorption experiments, the specific activities of the radioactive $CH_3{}^{131}I$ for all experiments were designed to be uniformly 180 Bq/mg at the commencement of the isotopic exchange, which serves as the reference time point.

**Dynamic Adsorption Test.** The dynamic adsorption experiment of radioactive $^{131}I_2$/ $CH_3{}^{131}I$ was conducted within a dynamic adsorption test platform equipped with an adsorption bed in the laboratory, as depicted in Figures S6 and Figure 3. The experimental procedure was as follows. After activating the powdered sample, a certain amount was loaded into a quartz column (6 mm I.D. ×150 mm) and sealed at both ends with quartz wool to prevent sample leakage. The quartz column was compressed to fill the gaps between the material and the column. An iodine chamber, boasting a volume of 785 L, primarily served to simulate an environment containing gaseous radioiodine. In preparation for the dynamic

adsorption test, the concentration of $CH_3{}^{131}I$ or $^{131}I_2$, temperature, and $NO_x$ concentration in the chamber were adjusted to the required conditions.

Typically, before each dynamic adsorption test, the iodine chamber is pre-set to the required temperature, $NO_x$ concentration, and $I_2/CH_3I$ concentration. The temperature within the chamber is regulated using a built-in temperature control module, which includes heating elements, internal temperature sensors, and a control circuit. These heating elements maintain the chamber temperature within ±1°C of the set point by continuously monitoring and adjusting as necessary.

Once the temperature reaches the set point, the internal pressure and $NO_x$ concentration are adjusted. In experiments not requiring $NO_x$, the chamber is directly exposed to ambient conditions to equate the internal pressure with the external atmosphere. When $NO_x$ is necessary, the chamber's pressure is adjusted using a vacuum pump to the target partial pressure—for example, to 80% of atmospheric pressure for a 20% $NO_x$ mixture. Subsequently, the $NO_x$ gas source is connected, allowing $NO_x$ to be introduced until the internal pressure matches the ambient pressure.

Finally, $I_2/CH_3I$ is generated and introduced into the chamber using a gaseous radioiodine generator. The amount of $I_2/CH_3I$ is controlled by the quantity of reagents and known yield to meet the required $I_2/CH_3I$ concentration. To validate the $I_2/CH_3I$ concentration, $I_2/CH_3I$ is sampled from the chamber and adsorbed onto an impregnated activated charcoal cartridge. The radioactivity of $^{131}I$ in the cartridge is measured using a HPGe gamma ray spectrometer, allowing for the estimation of the $I_2/CH_3I$ concentration. The estimated concentrations from these two methods are in close agreement with each other, with a relative deviation of less than 5%.

During the dynamic adsorption experiment, air containing $CH_3{}^{131}I$ or $^{131}I_2$ was continuously drawn into the adsorption column from the iodine chamber using an air pump and redirected back to the iodine chamber. The flow rate in this experiment was set at 0.6 L/min. The temperature of the adsorption column was controlled using a heating jacket. Throughout the adsorption process, a NaI(Tl) gamma ray detector continuously detected the gamma rays (364 keV) emitted from the adsorption column, enabling real-time monitoring of the adsorbed $^{131}I$ activity. After adsorption, the final activity of the sample tube was measured accurately using a HPGe gamma ray spectrometer.

**Analysis Method for HPGe Gamma Ray Spectrometer Measurements.** The HPGe gamma ray spectrometer has the advantage of low background and high measurement precision and is used to accurately measure the activity of $^{131}I$ in the adsorption column (Figure S7). The activity in the adsorption column is calculated using the following formula[9]:

$$A_{\mathrm{Ge}} = (N_1 - B_1)/(T_1 - \varepsilon_1)$$

Where $N_1$ is the total count of the peak of $^{131}I$ at 364 keV; $B_1$ is the background count of the peak at 364 keV by HPGe gamma ray spectrometer. $T_1$ is live time of measurement (typically between 1 to 10 min to ensure measurement precision better than 1%), and $\varepsilon_1$ is the detection efficiency of the HPGe gamma ray spectrometer for $^{131}I$ in the sample. The detection efficiency is influenced by the geometric shape, size, density of the adsorption material, and its relative geometric position with the detector. In this study, precise control of the relative geometric position between the adsorption material and the detector was ensured (as shown in Figure S8). The HPGe gamma ray spectrometer used in this study had been accurately calibrated using standard sources traceable to national standards, ensuring the accuracy and reliability of measurement results. Considering the relatively short half-life of $^{131}I$ (8.025 d), decay correction was applied using the following formula:

$$A_e = A_m e^{-\lambda(t_r - t_m)}$$

where $A_e$ is the corrected activity, $A_m$ is the measured activity, $\lambda$ is the decay constant of $^{131}I$ (0.0864 $d^{-1}$), $t_r$ is the reference time point of the radioactive $^{131}I_2$ or $CH_3{}^{131}I$, and $t_m$ is the beginning time of measurement (the measurement duration is short compared to the half-life of $^{131}I$, so the measurement start time is used as a representative).

After decay correction, the absorbed masses of $CH_3I$ or $I_2$, $m_a$, could be calculated from the corrected activity, $A_e$, by dividing by the specific activity of $CH_3I$ or $I_2$, $a_r$, (for all the adsorption experiments, the specific activities were designed to be uniformly 180 Bq/mg):

$$m_a = \frac{A_e}{a_r}$$

And the capture capability of the material, $c$, could be calculated from the absorbed masses, $m_a$, by dividing by the mass of the material used in the adsorption experiments, $m_m$:

$$c = \frac{m_a}{m_m}$$

**Analysis Method for NaI(Tl) Gamma Ray Detector Measurements.** While the HPGe gamma ray spectrometer offers high measurement accuracy, it is expensive and bulky, and samples can only be measured within its lead chamber. Therefore, measurements of $^{131}I$ activity could only be conducted on samples at the end of adsorption. To further investigate the dynamic adsorption process of $^{131}I_2/CH_3{}^{131}I$ throughout the entire adsorption process, this study set up a portable NaI(Tl) gamma ray detector close to the adsorption column to continuously monitor the activity of $^{131}I$ in the adsorption column in real-time (as shown in Figure S6).

The calculation formula for the activity of $^{131}I$ in the adsorption column is similar to that of the HPGe gamma ray spectrometer:

$$A_{\text{NaI(Tl)}} = (N_2 - B_2)/(T_2 - \varepsilon_2)$$

where $N_2$ is the total count of the peak of $^{131}I$ at 364 keV; $B_2$ is the background count of the peak at 364 keV by NaI(Tl) gamma ray detector. $T_2$ is the measurement time (in this study, continuous measurements were conducted with a 300 s cycle), and $\varepsilon_2$ is the detection efficiency of the NaI(Tl) gamma ray detector for $^{131}I$ in the sample, which was calculated by the last counting of NaI(Tl) and the activity of $^{131}I$ determined by HPGe gamma ray spectrometer.

Furthermore, it is crucial to account for the decay effects of $^{131}I$ in the measurements obtained from the NaI(T1) gamma ray spectrometer. To ensure accurate results, the aforementioned equation is uniformly adjusted to correct for the decay effects up to the reference time point of the radioactive $^{131}I_2$ or $CH_3{}^{131}I$.

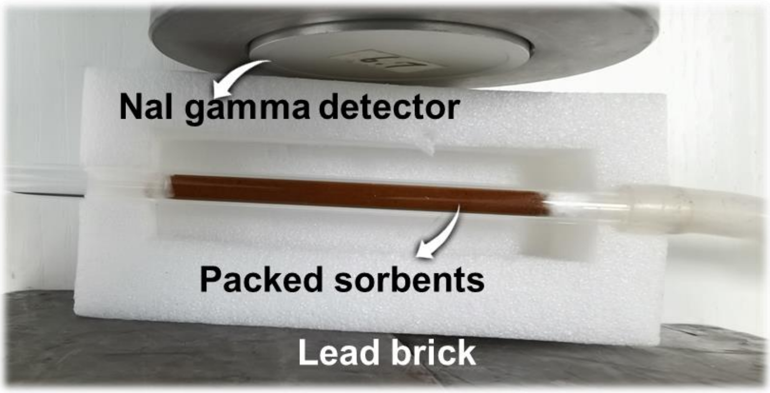


**Figure S6.** Photograph of NaI(Tl) gamma ray detector equipped with an adsorption bed.

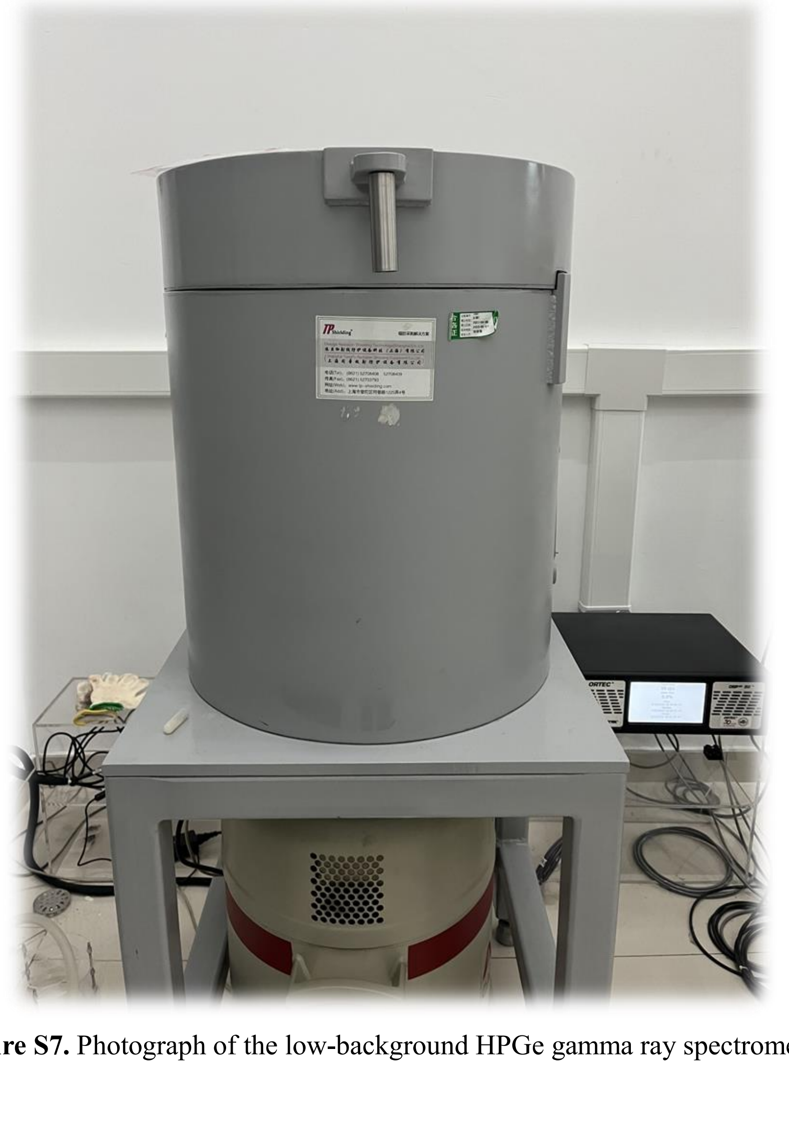


**Figure S7.** Photograph of the low-background HPGe gamma ray spectrometer.

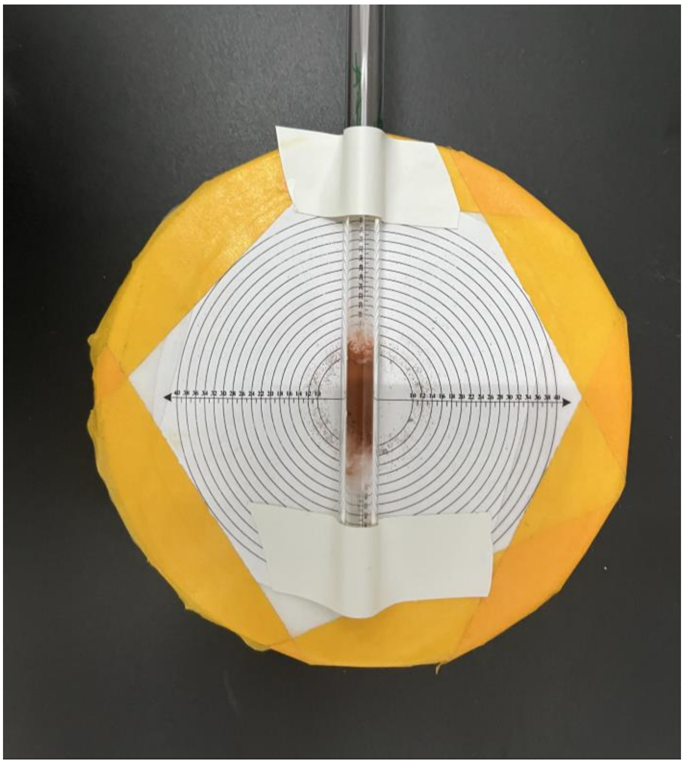

**Figure S8.** Photograph of the tested sample column on the low background HPGe gamma ray spectrometer.

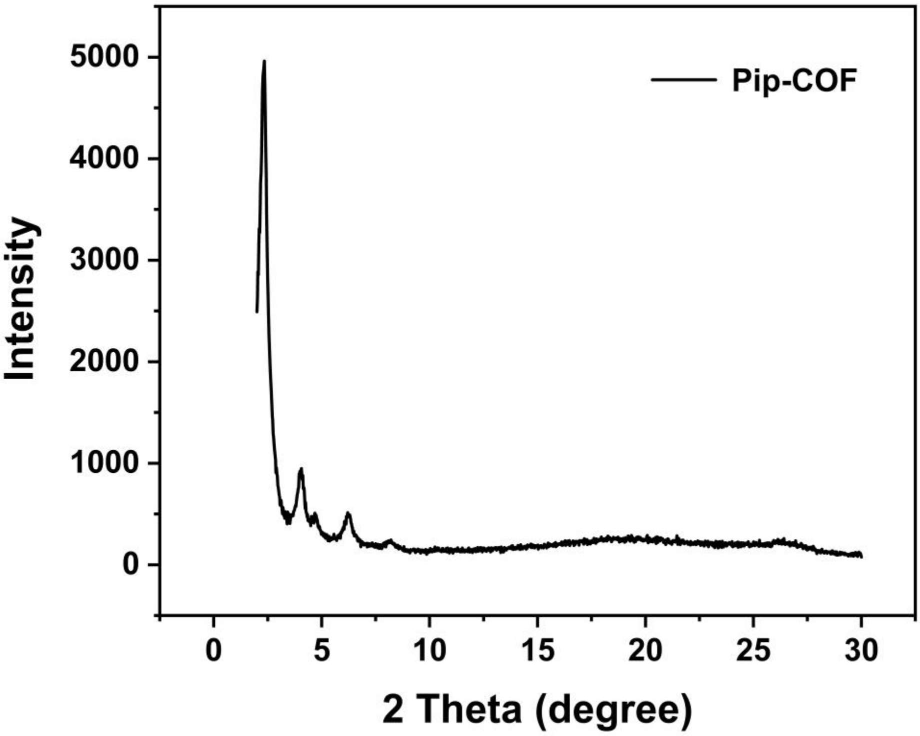


**Figure S9.** Experimental PXRD pattern of as-synthesized Pip-COF.

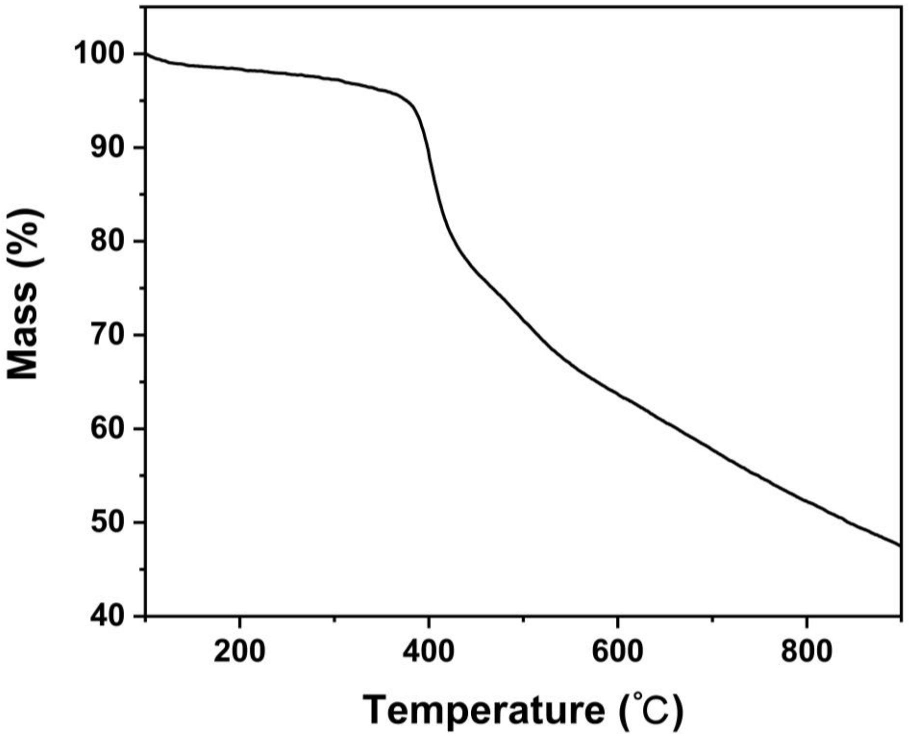


**Figure S10.** Thermogravimetric analysis (TGA) of Pip-COF.

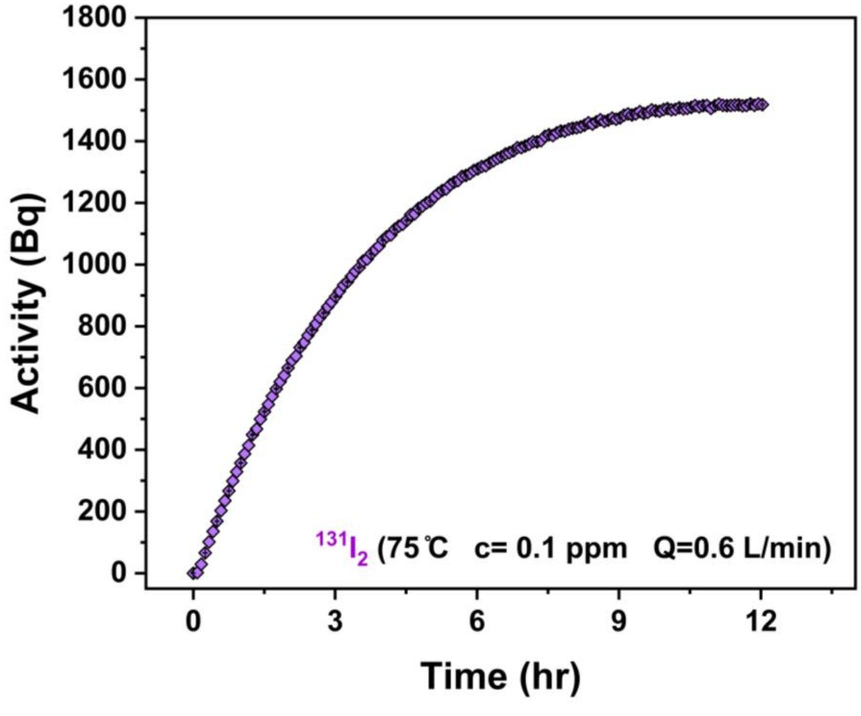


**Figure S11.** The column adsorption results for $^{131}I_2$ by Pip-COF under simulated off-gas system at 75℃.

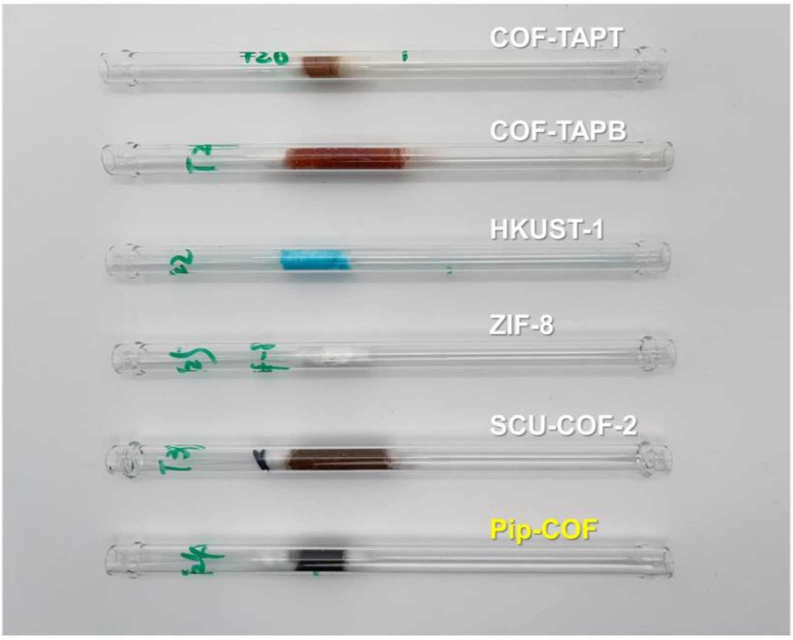


**Figure S12.** Photograph of the columns packed with Pip-COF and other reported column sorbents after adsorption of $CH_3^{131}I$.

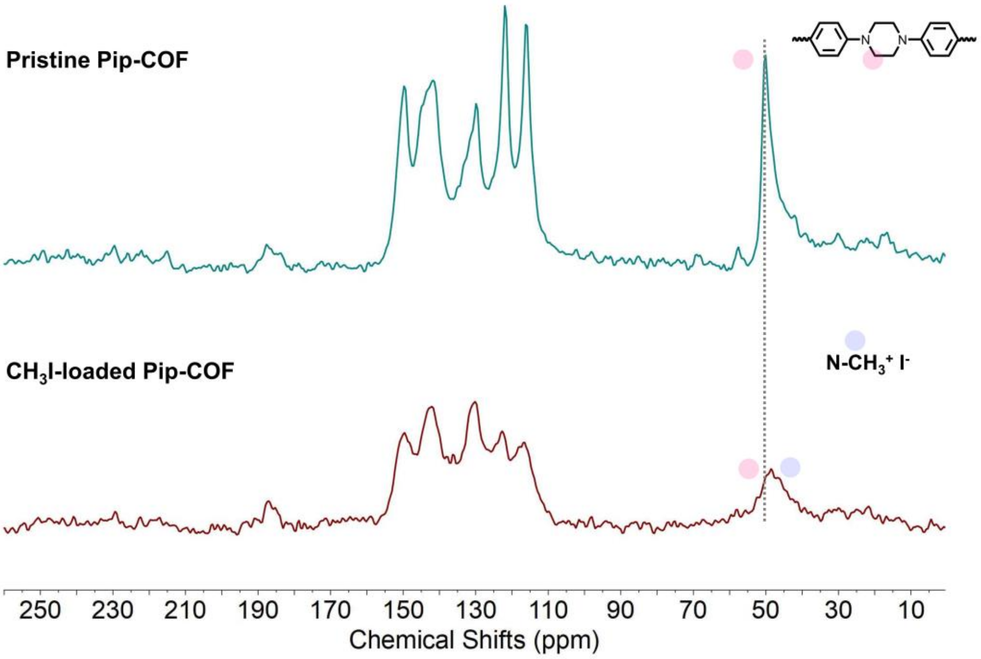


**Figure S13.** The solid-state $^{13}C$ NMR of pristine Pip-COF (green line) and $CH_3I$-loaded Pip-COF after adsorption (red line).

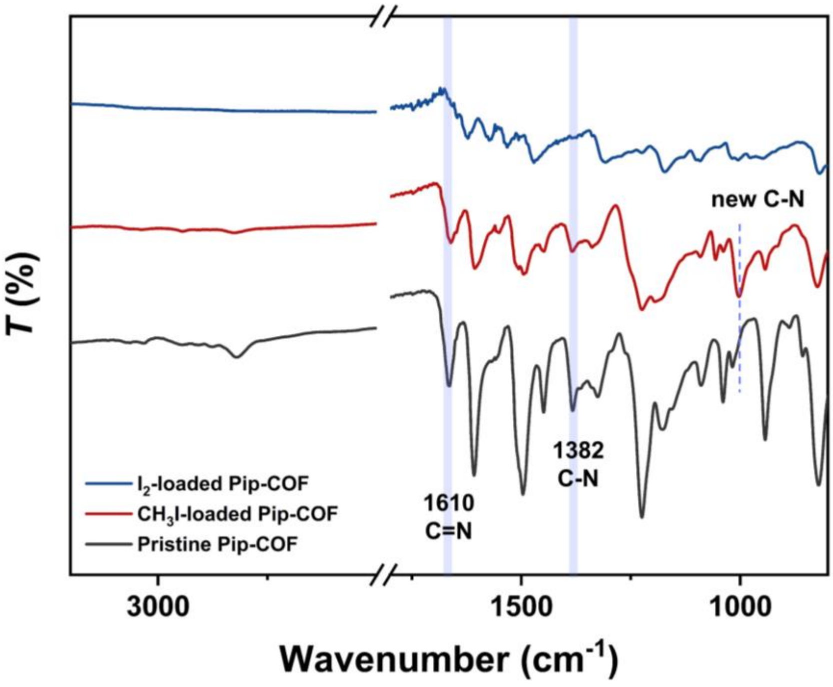


**Figure S14.** FT-IR spectra of pristine Pip-COF (black), $CH_3I$-loaded Pip-COF (red) and $I_2$-loaded Pip-COF (blue).

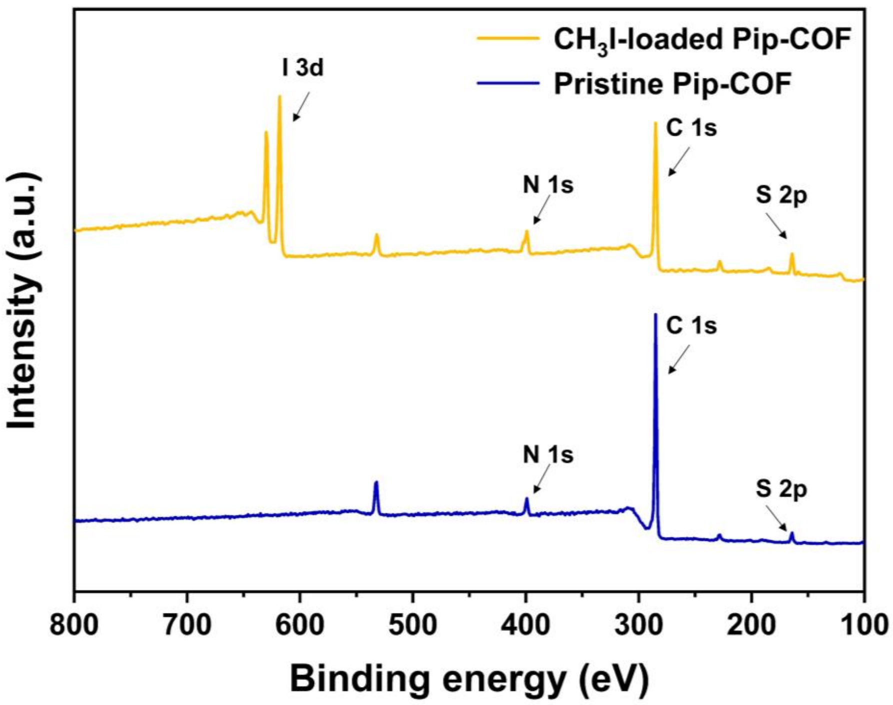


**Figure S15.** Full survey XPS spectra of pristine (blue) and $CH_3I$-loaded Pip-COF (yellow).

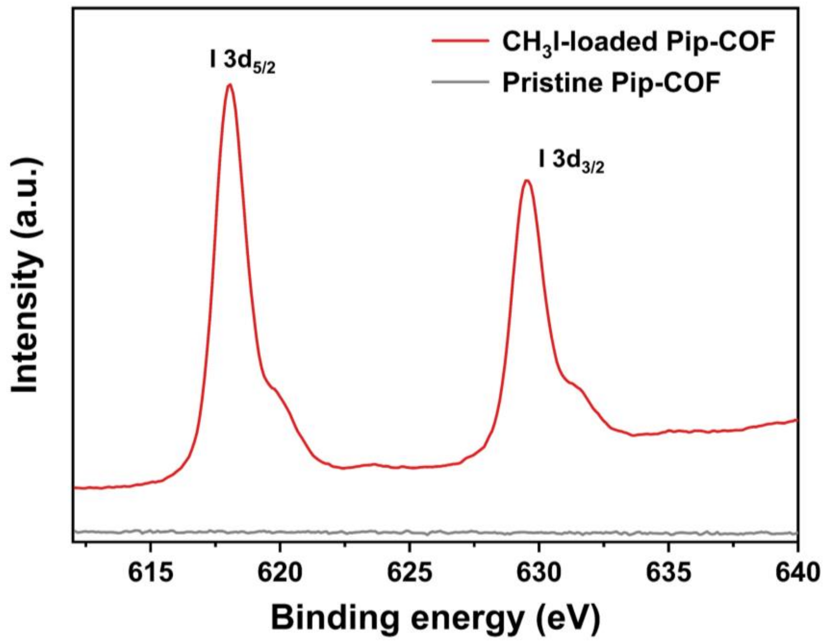


**Figure S16.** XPS spectra for I 3d of pristine (gray) and $CH_3I$-loaded Pip-COF (red).

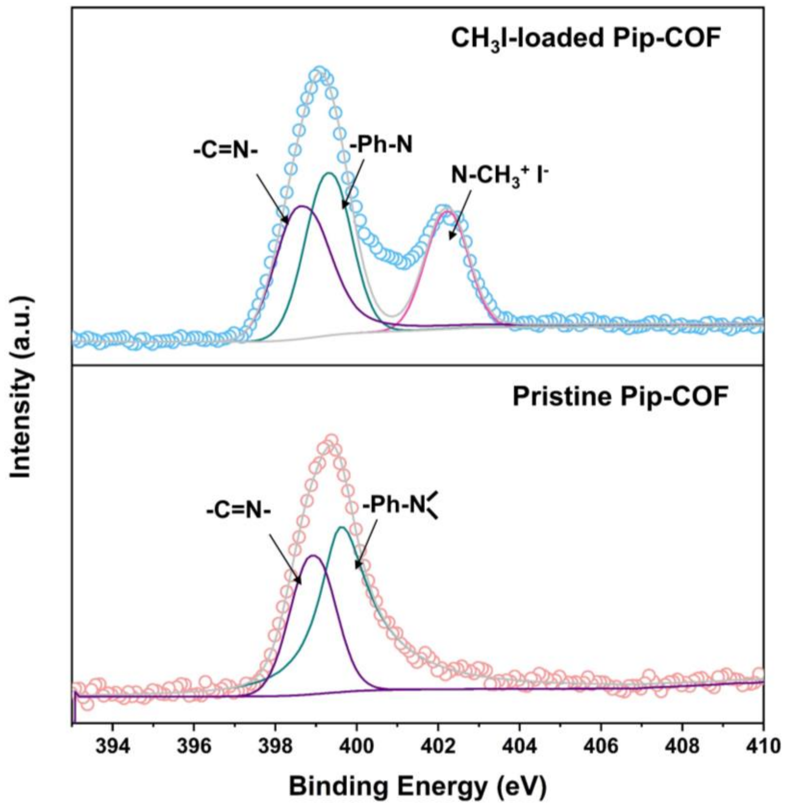


**Figure S17.** XPS spectra for N 1s of pristine and $CH_3I$-loaded Pip-COF.

**Table S1.** Comparison of dynamic adsorption capacity for $CH_3I$ by various column adsorbents under different conditions.

| Material type | Material | Adsorption condition | | | Adsorption capacity (g/g) | Ref. |
|---|---|---|---|---|---|---|
| | | Temperature (℃) | Concentration of $CH_3I$ (ppm) | Flow rate (mL/min) | | |
| **Zeolite** | $Ag^+$@13X | R.T. | 200000 | 3 | 0.45 | 10 |
| | $Ag^+$@MOR | R.T. | 200000 | 3 | 0.29 | 10 |
| | $Ag^+$@ZSM5 | R.T. | 200000 | 3 | 0.28 | 10 |
| | $Ag^0$@MOR | R.T. | 200000 | 3 | 0.25 | 10 |
| | HISL | R.T. | 200000 | 3 | 0.42 | 11 |
| **Composites** | HMTA@AC | R.T. | 200000 | 3 | 0.54 | 10 |
| | TED@AC | R.T. | 200000 | 3 | 0.52 | 10 |
| **MOF** | HKUST-1 | 35 | 1333 | 150 | 0.425 | 12 |
| | | R.T. | 5 | 600 | 0.03 | This work |
| | ZIF-8 | 35 | 1333 | 150 | 0.007 | 12 |
| | | R.T. | 5 | 600 | 0.002 | This work |
| | MIL-101-Cr-HMTA | R.T. | 200000 | 3 | 1.74 | 10 |
| | | R.T. | 50 | 400 | 0.51 | 13 |
| | MIL-101-Cr-TED | R.T. | 200000 | 3 | 1.6 | 10 |
| | MIL-120(Al) | 35 | 1333 | 150 | 0.164 | 12 |
| | MIL-53(Al) | 35 | 1333 | 150 | 0.127 | 12 |
| | UiO-66(Zr) | 35 | 1333 | 150 | 0.05 | 12 |
| | CAU-1(Al) | 35 | 1333 | 150 | 0.05 | 12 |
| | MIL-100Al) | 35 | 1333 | 150 | 0.013 | 12 |
| **COF** | COF-TAPT | R.T. | 200000 | 3 | 1.3 | 13 |
| | | R.T. | 50 | 400 | 0.39 | 13 |
| | | R.T. | 5 | 600 | 0.006 | This work |
| | COF-TAPB | R.T. | 200000 | 3 | 0.71 | 13 |
| | | R.T. | 50 | 400 | 0.12 | 13 |
| | | R.T. | 5 | 600 | 0.015 | This work |
| | SCU-COF-2 | R.T. | 200000 | 3 | 0.564 | 14 |
| | | R.T. | 50 | 400 | 0.08 | 13 |
| | | R.T. | 5 | 600 | 0.042 | This work |
| | iCOF-AB-50 | R.T. | 200000 | 3 | 0.62 | 13 |
| | | R.T. | 50 | 400 | 0.11 | 13 |
| | TFPA-TAPT | R.T. | 50 | 400 | 0.18 | 13 |
| | Pip-COF | R.T. | 5 | 600 | 0.078 | **This work** |
| | | 75 | 5 | 600 | 0.039 | **This work** |

**Table S2.** Atomic coordinates of the Pawley refined AA-stacking structural model of Pip-COF (space group *P6*/m, a = b = 44.866 Å, c = 3.412 Å, α = β = 90°, γ = 120°).

| Atom | x/a | y/b | z/c |
|---|---|---|---|
| C1 | -0.67052 | -0.30329 | -0.00006 |
| C2 | -0.70056 | -0.33719 | -0.00006 |
| S3 | -0.66609 | -0.26832 | 0.00001 |
| C4 | -0.63031 | -0.24457 | -0.00006 |
| C5 | -0.61178 | -0.26315 | -0.00006 |
| C6 | -0.61466 | -0.2066 | 0.00001 |
| N7 | -0.51547 | -0.03779 | 0.00003 |
| C8 | -0.4774 | -0.01451 | 0 |
| C9 | -0.53267 | -0.0203 | -0.0671 |
| C10 | -0.53058 | -0.0747 | 0.00005 |
| C11 | -0.50877 | -0.08979 | 0.00005 |
| C12 | -0.52387 | -0.1267 | 0.00005 |
| C13 | -0.56078 | -0.14849 | 0.00005 |
| C14 | -0.58259 | -0.13339 | 0.00005 |
| C15 | -0.56748 | -0.09648 | 0.00005 |
| N16 | -0.57696 | -0.18593 | 0.00001 |
| H17 | -0.58398 | -0.24927 | -0.00006 |
| H18 | -0.63092 | -0.1952 | 0.00006 |
| H19 | -0.46731 | -0.01658 | -0.27968 |
| H20 | -0.46605 | -0.02234 | 0.22798 |
| H21 | -0.55721 | -0.03503 | 0.07785 |
| H22 | -0.53855 | -0.02136 | -0.37546 |
| H23 | -0.48112 | -0.07346 | 0.00005 |
| H24 | -0.50755 | -0.13802 | 0.00005 |
| H25 | -0.61024 | -0.14973 | 0.00005 |
| H26 | -0.58381 | -0.08517 | 0.00005 |
| N7 | -0.96221 | -0.47768 | 0.00003 |
| C8 | -0.98549 | -0.46289 | 0 |
| C9 | -0.9797 | -0.51237 | -0.0671 |
| C10 | -0.9253 | -0.45588 | 0.00005 |
| C11 | -0.91021 | -0.41898 | 0.00005 |
| C12 | -0.8733 | -0.39717 | 0.00005 |
| C13 | -0.85151 | -0.41229 | 0.00005 |
| C14 | -0.86661 | -0.4492 | 0.00005 |
| C15 | -0.90352 | -0.471 | 0.00005 |
| N16 | -0.81407 | -0.39103 | 0.00001 |
| H19 | -0.98342 | -0.45073 | -0.27968 |
| H20 | -0.97766 | -0.44371 | 0.22798 |
| H21 | -0.96497 | -0.52218 | 0.07785 |

| | | | |
|---|---|---|---|
| H22 | -0.97864 | -0.51719 | -0.37546 |
| H23 | -0.92654 | -0.40766 | 0.00005 |
| H24 | -0.86198 | -0.36953 | 0.00005 |
| H25 | -0.85027 | -0.46051 | 0.00005 |
| H26 | -0.91483 | -0.49864 | 0.00005 |
| C1 | -0.69671 | -0.36723 | -0.00006 |
| C2 | -0.66281 | -0.36337 | -0.00006 |
| S3 | -0.73168 | -0.39777 | 0.00001 |
| C4 | -0.75543 | -0.38574 | -0.00006 |
| C5 | -0.73685 | -0.34863 | -0.00006 |
| C6 | -0.7934 | -0.40806 | 0.00001 |
| H17 | -0.75073 | -0.33471 | -0.00006 |
| H18 | -0.8048 | -0.43572 | 0.00006 |
| N7 | -0.52232 | -0.48453 | 0.00003 |
| C8 | -0.53711 | -0.5226 | 0 |
| C9 | -0.48763 | -0.46733 | -0.0671 |
| C10 | -0.54412 | -0.46942 | 0.00005 |
| C11 | -0.58102 | -0.49123 | 0.00005 |
| C12 | -0.60283 | -0.47613 | 0.00005 |
| C13 | -0.58771 | -0.43922 | 0.00005 |
| C14 | -0.5508 | -0.41741 | 0.00005 |
| C15 | -0.529 | -0.43252 | 0.00005 |
| N16 | -0.60897 | -0.42304 | 0.00001 |
| H19 | -0.54927 | -0.53269 | -0.27968 |
| H20 | -0.55629 | -0.53395 | 0.22798 |
| H21 | -0.47782 | -0.44279 | 0.07785 |
| H22 | -0.48281 | -0.46145 | -0.37546 |
| H23 | -0.59234 | -0.51888 | 0.00005 |
| H24 | -0.63047 | -0.49245 | 0.00005 |
| H25 | -0.53949 | -0.38976 | 0.00005 |
| H26 | -0.50136 | -0.41619 | 0.00005 |
| C1 | -0.63277 | -0.32948 | -0.00006 |
| C2 | -0.63663 | -0.29944 | -0.00006 |
| S3 | -0.60223 | -0.33391 | 0.00001 |
| C4 | -0.61426 | -0.36969 | -0.00006 |
| C5 | -0.65137 | -0.38822 | -0.00006 |
| C6 | -0.59194 | -0.38534 | 0.00001 |
| H17 | -0.66529 | -0.41602 | -0.00006 |
| H18 | -0.56428 | -0.36908 | 0.00006 |
| N7 | -0.48453 | 0.03779 | -0.00003 |
| C8 | -0.5226 | 0.01451 | 0 |
| C9 | -0.46733 | 0.0203 | 0.0671 |
| C10 | -0.46942 | 0.0747 | -0.00005 |

| | | | |
|---|---|---|---|
| C11 | -0.49123 | 0.08979 | -0.00005 |
| C12 | -0.47613 | 0.1267 | -0.00005 |
| C13 | -0.43922 | 0.14849 | -0.00005 |
| C14 | -0.41741 | 0.13339 | -0.00005 |
| C15 | -0.43252 | 0.09648 | -0.00005 |
| N16 | -0.42304 | 0.18593 | -0.00001 |
| H19 | -0.53269 | 0.01658 | 0.27968 |
| H20 | -0.53395 | 0.02234 | -0.22798 |
| H21 | -0.44279 | 0.03503 | -0.07785 |
| H22 | -0.46145 | 0.02136 | 0.37546 |
| H23 | -0.51888 | 0.07346 | -0.00005 |
| H24 | -0.49245 | 0.13802 | -0.00005 |
| H25 | -0.38976 | 0.14973 | -0.00005 |
| H26 | -0.41619 | 0.08517 | -0.00005 |
| C1 | -0.32948 | 0.30329 | 0.00006 |
| C2 | -0.29944 | 0.33719 | 0.00006 |
| S3 | -0.33391 | 0.26832 | -0.00001 |
| C4 | -0.36969 | 0.24457 | 0.00006 |
| C5 | -0.38822 | 0.26315 | 0.00006 |
| C6 | -0.38534 | 0.2066 | -0.00001 |
| H17 | -0.41602 | 0.24927 | 0.00006 |
| H18 | -0.36908 | 0.1952 | -0.00006 |
| N7 | -1.03779 | -0.52232 | -0.00003 |
| C8 | -1.01451 | -0.53711 | 0 |
| C9 | -1.0203 | -0.48763 | 0.0671 |
| C10 | -1.0747 | -0.54412 | -0.00005 |
| C11 | -1.08979 | -0.58102 | -0.00005 |
| C12 | -1.1267 | -0.60283 | -0.00005 |
| C13 | -1.14849 | -0.58771 | -0.00005 |
| C14 | -1.13339 | -0.5508 | -0.00005 |
| C15 | -1.09648 | -0.529 | -0.00005 |
| N16 | -1.18593 | -0.60897 | -0.00001 |
| H19 | -1.01658 | -0.54927 | 0.27968 |
| H20 | -1.02234 | -0.55629 | -0.22798 |
| H21 | -1.03503 | -0.47782 | -0.07785 |
| H22 | -1.02136 | -0.48281 | 0.37546 |
| H23 | -1.07346 | -0.59234 | -0.00005 |
| H24 | -1.13802 | -0.63047 | -0.00005 |
| H25 | -1.14973 | -0.53949 | -0.00005 |
| H26 | -1.08517 | -0.50136 | -0.00005 |
| C1 | -1.30329 | -0.63277 | 0.00006 |
| C2 | -1.33719 | -0.63663 | 0.00006 |
| S3 | -1.26832 | -0.60223 | -0.00001 |

| | | | |
|---|---|---|---|
| C4 | -1.24457 | -0.61426 | 0.00006 |
| C5 | -1.26315 | -0.65137 | 0.00006 |
| C6 | -1.2066 | -0.59194 | -0.00001 |
| H17 | -1.24927 | -0.66529 | 0.00006 |
| H18 | -1.1952 | -0.56428 | -0.00006 |
| N7 | -0.47768 | -0.51547 | -0.00003 |
| C8 | -0.46289 | -0.4774 | 0 |
| C9 | -0.51237 | -0.53267 | 0.0671 |
| C10 | -0.45588 | -0.53058 | -0.00005 |
| C11 | -0.41898 | -0.50877 | -0.00005 |
| C12 | -0.39717 | -0.52387 | -0.00005 |
| C13 | -0.41229 | -0.56078 | -0.00005 |
| C14 | -0.4492 | -0.58259 | -0.00005 |
| C15 | -0.471 | -0.56748 | -0.00005 |
| N16 | -0.39103 | -0.57696 | -0.00001 |
| H19 | -0.45073 | -0.46731 | 0.27968 |
| H20 | -0.44371 | -0.46605 | -0.22798 |
| H21 | -0.52218 | -0.55721 | -0.07785 |
| H22 | -0.51719 | -0.53855 | 0.37546 |
| H23 | -0.40766 | -0.48112 | -0.00005 |
| H24 | -0.36953 | -0.50755 | -0.00005 |
| H25 | -0.46051 | -0.61024 | -0.00005 |
| H26 | -0.49864 | -0.58381 | -0.00005 |
| C1 | -0.36723 | -0.67052 | 0.00006 |
| C2 | -0.36337 | -0.70056 | 0.00006 |
| S3 | -0.39777 | -0.66609 | -0.00001 |
| C4 | -0.38574 | -0.63031 | 0.00006 |
| C5 | -0.34863 | -0.61178 | 0.00006 |
| C6 | -0.40806 | -0.61466 | -0.00001 |
| H17 | -0.33471 | -0.58398 | 0.00006 |
| H18 | -0.43572 | -0.63092 | -0.00006 |
| C1 | -1.69671 | -1.36723 | -0.00006 |
| C2 | -1.66281 | -1.36337 | -0.00006 |
| S3 | -1.73168 | -1.39777 | 0.00001 |
| C4 | -1.75543 | -1.38574 | -0.00006 |
| C5 | -1.73685 | -1.34863 | -0.00006 |
| C6 | -1.7934 | -1.40806 | 0.00001 |
| N7 | -1.96221 | -1.47768 | 0.00003 |
| C8 | -1.98549 | -1.46289 | 0 |
| C9 | -1.9797 | -1.51237 | -0.0671 |
| C10 | -1.9253 | -1.45588 | 0.00005 |
| C11 | -1.91021 | -1.41898 | 0.00005 |
| C12 | -1.8733 | -1.39717 | 0.00005 |

| | | | |
|---|---|---|---|
| C13 | -1.85151 | -1.41229 | 0.00005 |
| C14 | -1.86661 | -1.4492 | 0.00005 |
| C15 | -1.90352 | -1.471 | 0.00005 |
| N16 | -1.81407 | -1.39103 | 0.00001 |
| H17 | -1.75073 | -1.33471 | -0.00006 |
| H18 | -1.8048 | -1.43572 | 0.00006 |
| H19 | -1.98342 | -1.45073 | -0.27968 |
| H20 | -1.97766 | -1.44371 | 0.22798 |
| H21 | -1.96497 | -1.52218 | 0.07785 |
| H22 | -1.97864 | -1.51719 | -0.37546 |
| H23 | -1.92654 | -1.40766 | 0.00005 |
| H24 | -1.86198 | -1.36953 | 0.00005 |
| H25 | -1.85027 | -1.46051 | 0.00005 |
| H26 | -1.91483 | -1.49864 | 0.00005 |
| N7 | -1.52232 | -1.48453 | 0.00003 |
| C8 | -1.53711 | -1.5226 | 0 |
| C9 | -1.48763 | -1.46733 | -0.0671 |
| C10 | -1.54412 | -1.46942 | 0.00005 |
| C11 | -1.58102 | -1.49123 | 0.00005 |
| C12 | -1.60283 | -1.47613 | 0.00005 |
| C13 | -1.58771 | -1.43922 | 0.00005 |
| C14 | -1.5508 | -1.41741 | 0.00005 |
| C15 | -1.529 | -1.43252 | 0.00005 |
| N16 | -1.60897 | -1.42304 | 0.00001 |
| H19 | -1.54927 | -1.53269 | -0.27968 |
| H20 | -1.55629 | -1.53395 | 0.22798 |
| H21 | -1.47782 | -1.44279 | 0.07785 |
| H22 | -1.48281 | -1.46145 | -0.37546 |
| H23 | -1.59234 | -1.51888 | 0.00005 |
| H24 | -1.63047 | -1.49245 | 0.00005 |
| H25 | -1.53949 | -1.38976 | 0.00005 |
| H26 | -1.50136 | -1.41619 | 0.00005 |
| C1 | -1.63277 | -1.32948 | -0.00006 |
| C2 | -1.63663 | -1.29944 | -0.00006 |
| S3 | -1.60223 | -1.33391 | 0.00001 |
| C4 | -1.61426 | -1.36969 | -0.00006 |
| C5 | -1.65137 | -1.38822 | -0.00006 |
| C6 | -1.59194 | -1.38534 | 0.00001 |
| H17 | -1.66529 | -1.41602 | -0.00006 |
| H18 | -1.56428 | -1.36908 | 0.00006 |
| N7 | -1.51547 | -1.03779 | 0.00003 |
| C8 | -1.4774 | -1.01451 | 0 |
| C9 | -1.53267 | -1.0203 | -0.0671 |

| | | | |
|---|---|---|---|
| C10 | -1.53058 | -1.0747 | 0.00005 |
| C11 | -1.50877 | -1.08979 | 0.00005 |
| C12 | -1.52387 | -1.1267 | 0.00005 |
| C13 | -1.56078 | -1.14849 | 0.00005 |
| C14 | -1.58259 | -1.13339 | 0.00005 |
| C15 | -1.56748 | -1.09648 | 0.00005 |
| N16 | -1.57696 | -1.18593 | 0.00001 |
| H19 | -1.46731 | -1.01658 | -0.27968 |
| H20 | -1.46605 | -1.02234 | 0.22798 |
| H21 | -1.55721 | -1.03503 | 0.07785 |
| H22 | -1.53855 | -1.02136 | -0.37546 |
| H23 | -1.48112 | -1.07346 | 0.00005 |
| H24 | -1.50755 | -1.13802 | 0.00005 |
| H25 | -1.61024 | -1.14973 | 0.00005 |
| H26 | -1.58381 | -1.08517 | 0.00005 |
| C1 | -1.67052 | -1.30329 | -0.00006 |
| C2 | -1.70056 | -1.33719 | -0.00006 |
| S3 | -1.66609 | -1.26832 | 0.00001 |
| C4 | -1.63031 | -1.24457 | -0.00006 |
| C5 | -1.61178 | -1.26315 | -0.00006 |
| C6 | -1.61466 | -1.2066 | 0.00001 |
| H17 | -1.58398 | -1.24927 | -0.00006 |
| H18 | -1.63092 | -1.1952 | 0.00006 |
| N7 | -2.03779 | -1.52232 | -0.00003 |
| C8 | -2.01451 | -1.53711 | 0 |
| C9 | -2.0203 | -1.48763 | 0.0671 |
| C10 | -2.0747 | -1.54412 | -0.00005 |
| C11 | -2.08979 | -1.58102 | -0.00005 |
| C12 | -2.1267 | -1.60283 | -0.00005 |
| C13 | -2.14849 | -1.58771 | -0.00005 |
| C14 | -2.13339 | -1.5508 | -0.00005 |
| C15 | -2.09648 | -1.529 | -0.00005 |
| N16 | -2.18593 | -1.60897 | -0.00001 |
| H19 | -2.01658 | -1.54927 | 0.27968 |
| H20 | -2.02234 | -1.55629 | -0.22798 |
| H21 | -2.03503 | -1.47782 | -0.07785 |
| H22 | -2.02136 | -1.48281 | 0.37546 |
| H23 | -2.07346 | -1.59234 | -0.00005 |
| H24 | -2.13802 | -1.63047 | -0.00005 |
| H25 | -2.14973 | -1.53949 | -0.00005 |
| H26 | -2.08517 | -1.50136 | -0.00005 |
| C1 | -2.30329 | -1.63277 | 0.00006 |
| C2 | -2.33719 | -1.63663 | 0.00006 |

| | | | |
|---|---|---|---|
| S3 | -2.26832 | -1.60223 | -0.00001 |
| C4 | -2.24457 | -1.61426 | 0.00006 |
| C5 | -2.26315 | -1.65137 | 0.00006 |
| C6 | -2.2066 | -1.59194 | -0.00001 |
| H17 | -2.24927 | -1.66529 | 0.00006 |
| H18 | -2.1952 | -1.56428 | -0.00006 |
| N7 | -1.47768 | -1.51547 | -0.00003 |
| C8 | -1.46289 | -1.4774 | 0 |
| C9 | -1.51237 | -1.53267 | 0.0671 |
| C10 | -1.45588 | -1.53058 | -0.00005 |
| C11 | -1.41898 | -1.50877 | -0.00005 |
| C12 | -1.39717 | -1.52387 | -0.00005 |
| C13 | -1.41229 | -1.56078 | -0.00005 |
| C14 | -1.4492 | -1.58259 | -0.00005 |
| C15 | -1.471 | -1.56748 | -0.00005 |
| N16 | -1.39103 | -1.57696 | -0.00001 |
| H19 | -1.45073 | -1.46731 | 0.27968 |
| H20 | -1.44371 | -1.46605 | -0.22798 |
| H21 | -1.52218 | -1.55721 | -0.07785 |
| H22 | -1.51719 | -1.53855 | 0.37546 |
| H23 | -1.40766 | -1.48112 | -0.00005 |
| H24 | -1.36953 | -1.50755 | -0.00005 |
| H25 | -1.46051 | -1.61024 | -0.00005 |
| H26 | -1.49864 | -1.58381 | -0.00005 |
| C1 | -1.36723 | -1.67052 | 0.00006 |
| C2 | -1.36337 | -1.70056 | 0.00006 |
| S3 | -1.39777 | -1.66609 | -0.00001 |
| C4 | -1.38574 | -1.63031 | 0.00006 |
| C5 | -1.34863 | -1.61178 | 0.00006 |
| C6 | -1.40806 | -1.61466 | -0.00001 |
| H17 | -1.33471 | -1.58398 | 0.00006 |
| H18 | -1.43572 | -1.63092 | -0.00006 |
| N7 | -1.48453 | -0.96221 | -0.00003 |
| C8 | -1.5226 | -0.98549 | 0 |
| C9 | -1.46733 | -0.9797 | 0.0671 |
| C10 | -1.46942 | -0.9253 | -0.00005 |
| C11 | -1.49123 | -0.91021 | -0.00005 |
| C12 | -1.47613 | -0.8733 | -0.00005 |
| C13 | -1.43922 | -0.85151 | -0.00005 |
| C14 | -1.41741 | -0.86661 | -0.00005 |
| C15 | -1.43252 | -0.90352 | -0.00005 |
| N16 | -1.42304 | -0.81407 | -0.00001 |
| H19 | -1.53269 | -0.98342 | 0.27968 |

| | | | |
|---|---|---|---|
| H20 | -1.53395 | -0.97766 | -0.22798 |
| H21 | -1.44279 | -0.96497 | -0.07785 |
| H22 | -1.46145 | -0.97864 | 0.37546 |
| H23 | -1.51888 | -0.92654 | -0.00005 |
| H24 | -1.49245 | -0.86198 | -0.00005 |
| H25 | -1.38976 | -0.85027 | -0.00005 |
| H26 | -1.41619 | -0.91483 | -0.00005 |
| C1 | -1.32948 | -0.69671 | 0.00006 |
| C2 | -1.29944 | -0.66281 | 0.00006 |
| S3 | -1.33391 | -0.73168 | -0.00001 |
| C4 | -1.36969 | -0.75543 | 0.00006 |
| C5 | -1.38822 | -0.73685 | 0.00006 |
| C6 | -1.38534 | -0.7934 | -0.00001 |
| H17 | -1.41602 | -0.75073 | 0.00006 |
| H18 | -1.36908 | -0.8048 | -0.00006 |
| N7 | -1.47768 | -0.51547 | -0.00003 |
| C8 | -1.46289 | -0.4774 | 0 |
| C9 | -1.51237 | -0.53267 | 0.0671 |
| C10 | -1.45588 | -0.53058 | -0.00005 |
| C11 | -1.41898 | -0.50877 | -0.00005 |
| C12 | -1.39717 | -0.52387 | -0.00005 |
| C13 | -1.41229 | -0.56078 | -0.00005 |
| C14 | -1.4492 | -0.58259 | -0.00005 |
| C15 | -1.471 | -0.56748 | -0.00005 |
| N16 | -1.39103 | -0.57696 | -0.00001 |
| H19 | -1.45073 | -0.46731 | 0.27968 |
| H20 | -1.44371 | -0.46605 | -0.22798 |
| H21 | -1.52218 | -0.55721 | -0.07785 |
| H22 | -1.51719 | -0.53855 | 0.37546 |
| H23 | -1.40766 | -0.48112 | -0.00005 |
| H24 | -1.36953 | -0.50755 | -0.00005 |
| H25 | -1.46051 | -0.61024 | -0.00005 |
| H26 | -1.49864 | -0.58381 | -0.00005 |
| C1 | -1.36723 | -0.67052 | 0.00006 |
| C2 | -1.36337 | -0.70056 | 0.00006 |
| S3 | -1.39777 | -0.66609 | -0.00001 |
| C4 | -1.38574 | -0.63031 | 0.00006 |
| C5 | -1.34863 | -0.61178 | 0.00006 |
| C6 | -1.40806 | -0.61466 | -0.00001 |
| H17 | -1.33471 | -0.58398 | 0.00006 |
| H18 | -1.43572 | -0.63092 | -0.00006 |
| N7 | -1.52232 | -0.48453 | 0.00003 |
| C8 | -1.53711 | -0.5226 | 0 |

| | | | |
|---|---|---|---|
| C9 | -1.48763 | -0.46733 | -0.0671 |
| C10 | -1.54412 | -0.46942 | 0.00005 |
| C11 | -1.58102 | -0.49123 | 0.00005 |
| C12 | -1.60283 | -0.47613 | 0.00005 |
| C13 | -1.58771 | -0.43922 | 0.00005 |
| C14 | -1.5508 | -0.41741 | 0.00005 |
| C15 | -1.529 | -0.43252 | 0.00005 |
| N16 | -1.60897 | -0.42304 | 0.00001 |
| H19 | -1.54927 | -0.53269 | -0.27968 |
| H20 | -1.55629 | -0.53395 | 0.22798 |
| H21 | -1.47782 | -0.44279 | 0.07785 |
| H22 | -1.48281 | -0.46145 | -0.37546 |
| H23 | -1.59234 | -0.51888 | 0.00005 |
| H24 | -1.63047 | -0.49245 | 0.00005 |
| H25 | -1.53949 | -0.38976 | 0.00005 |
| H26 | -1.50136 | -0.41619 | 0.00005 |
| C1 | -1.63277 | -0.32948 | -0.00006 |
| C2 | -1.63663 | -0.29944 | -0.00006 |
| S3 | -1.60223 | -0.33391 | 0.00001 |
| C4 | -1.61426 | -0.36969 | -0.00006 |
| C5 | -1.65137 | -0.38822 | -0.00006 |
| C6 | -1.59194 | -0.38534 | 0.00001 |
| H17 | -1.66529 | -0.41602 | -0.00006 |
| H18 | -1.56428 | -0.36908 | 0.00006 |
| C1 | -1.67052 | -0.30329 | -0.00006 |
| C1 | -1.32948 | -1.69671 | 0.00006 |
| C1 | -0.32948 | -0.69671 | 0.00006 |
| C1 | -0.30329 | 0.36723 | 0.00006 |
| C1 | -2.36723 | -1.67052 | 0.00006 |
| C2 | -1.66281 | -0.36337 | -0.00006 |
| C2 | -2.29944 | -1.66281 | 0.00006 |
| C2 | -1.33719 | -1.63663 | 0.00006 |
| C2 | -0.33719 | -0.63663 | 0.00006 |
| C2 | -0.36337 | 0.29944 | 0.00006 |
| C5 | -1.61178 | -0.26315 | -0.00006 |
| C5 | -1.38822 | -1.73685 | 0.00006 |
| C5 | -0.38822 | -0.73685 | 0.00006 |
| C5 | -0.26315 | 0.34863 | 0.00006 |
| C5 | -2.34863 | -1.61178 | 0.00006 |

**References**


(1) Frisch, M. J.; et al. Gaussian 09, Revision E.01, Gaussian, Inc., Wallingford CT, **2009**.

(2) Perdew, J. P.; Ernzerhof, M.; Burke, K. Rationale for mixing exact exchange with density functional approximations. *J. Chem. Phys*. **1996**, *105* (22), 9982-9985.

(3) F. Weigend, R. Ahlrichs. Balanced basis sets of split valence, triple zeta valence and quadruple zeta valence quality for H to Rn: Design and assessment of accuracy. *Phys. Chem. Chem. Phys*. **2005**, *7*, 3297-3305.

(4) Chai, J. D.; Head-Gordon, M. Long-range corrected hybrid density functionals with damped atom-atom dispersion corrections. *Phys. Chem. Chem. Phys*. **2008**, *10*, 6615-6620.

(5) Chai, J. D.; Head-Gordon, M. Systematic optimization of long-range corrected hybrid density functionals. *J. Chem. Phys*. **2008**, *128*, 084106.

(6) Boys, S. F.; Bernardi, F. The calculation of small molecular interactions by the differences of separate total energies. Some procedures with reduced errors. *Mol. Phys*. **2002**, *100* (1), 73.

(7) Chino, M.; Nakayama, H.; Nagai, H.; Terada, H.; Katata, G.; Yamazawa, H. Preliminary Estimation of Release Amounts of $^{131}$I and $^{137}$Cs Accidentally Discharged from the Fukushima Daiichi Nuclear Power Plant into the Atmosphere. *J. Nucl. Sci. Technol*. **2011**, *48* (7), 1129-1134.

(8) Zhao, C.; Tang, F.; He, L.; Xu, Y.; Lu, X. Development of A Calibration System for Airborne $^{131}$I Monitoring Devices. *Appl. Radiat. Isot*. **2016**, *109*, 460-464.

(9) Zhao, C.; Chen, Y.; Liu, J. Development of A High-Efficiency Gaseous Elemental Radioiodine Generator. *Rev. Sci. Instrum*. **2022**, *93*, 043307.

(10) Li, B.; Dong, X.; Wang, H.; Ma, D.; Tan, K.; Jensen, S.; Deibert, B. J.; Butler, J.; Cure, J.; Shi, Z.; et al. Capture of organic iodides from nuclear waste by metal-organic framework-based molecular traps. *Nat. Commun.* **2017**, *8*, 485.

(11) Pham, T. C. T.; Docao, S.; Hwang, I. C.; Song, M. K.; Choi, D. Y.; Moon, D.; Oleynikov, P.; Yoon, K. B. Capture of iodine and organic iodides using silica zeolites and the semiconductor behaviour of iodine in a silica zeolite. *Energy Environ. Sci*. **2016**, *9*, 1050-1062.

(12) Chebbi, M.; Azambre, B.; Volkringer, C.; Loiseau, T. Dynamic sorption properties of metal-organic frameworks for the capture of methyl iodide. *Micro. Meso. Mater*., **2018**, *259*, 244-254.

(13) Xie, Y.; Pan, T.; Lei, Q.; Chen, C.; Dong, X.; Yuan, Y.; Maksoud, W. A.; Zhao, L.; Cavallo, L.; Pinnau, I.; Han, Y. Efficient and simultaneous capture of iodine and methyl iodide achieved by a covalent organic framework. *Nat. Commun.* **2022**, *13*, 2878.

(14) He, L.; Li, B.; Ma, Z.; Chen, L.; Gong, S.; Zhang, M.; Bai, Y.; Guo, Q.; Wu, F.; Zhao, F.; Li, J.; Zhang, D.; Sheng, D.; Dai, X.; Chen, L.; Shu, J.; Chai, Z.; Wang, S.; *Sci. China Chem.* **2023**, *66*, 783-790.

(15) Lu, T.; Chen, F. *J. Comput. Chem.* **2012**, *33*, 580-592.

**Appendix: The acronyms of chemicals or sorbent materials mentioned in main text.**

1. Pip = piperazine
2. BANPZ = 1,4-bis(4-aminophenyl)piperazine
3. BTT = benzobis[1,2-B:3,4-B':5,6-B']trithiophene-2,5,8-trialdehyde
4. TAPB = 1,3,5-tris(4-aminophenyl)benzene
5. TAPT = 2,4,6-tri(4-aminophenyl)-1,3,5-triazine
6. SCU = Soochow University
7. HKUST = Hong Kong University of Science and Technology
8. ZIF = zeolitic imidazolate frameworks
